# Process-property correlations in laser-induced graphene electrodes for electrochemical sensing

**DOI:** 10.1007/s00604-021-04792-3

**Published:** 2021-04-07

**Authors:** Arne Behrent, Christian Griesche, Paul Sippel, Antje J. Baeumner

**Affiliations:** Institute of Analytical Chemistry, Chemo- and Biosensors, University of Regensburg, Regensburg, Germany

**Keywords:** Laser-induced graphene, Porous carbon, Fabrication parameters, Chemical sensor, Voltammetry

## Abstract

Laser-induced graphene (LIG) has emerged as a promising electrode material for electrochemical point-of-care diagnostics. LIG offers a large specific surface area and excellent electron transfer at low-cost in a binder-free and rapid fabrication process that lends itself well to mass production outside of the cleanroom. Various LIG micromorphologies can be generated when altering the energy input parameters, and it was investigated here which impact this has on their electroanalytical characteristics and performance. Energy input is well controlled by the laser power, scribing speed, and laser pulse density. Once the threshold of required energy input is reached a broad spectrum of conditions leads to LIG with micromorphologies ranging from delicate irregular brush structures obtained at fast, high energy input, to smoother and more wall like albeit still porous materials. Only a fraction of these LIG structures provided high conductance which is required for appropriate electroanalytical performance. Here, it was found that low, frequent energy input provided the best electroanalytical material, i.e., low levels of power and speed in combination with high spatial pulse density. For example, the sensitivity for the reduction of K_3_[Fe(CN)_6_] was increased almost 2-fold by changing fabrication parameters from 60% power and 100% speed to 1% power and 10% speed. These general findings can be translated to any LIG fabrication process independent of devices used. The simple fabrication process of LIG electrodes, their good electroanalytical performance as demonstrated here with a variety of (bio)analytically relevant molecules including ascorbic acid, dopamine, uric acid, p-nitrophenol, and paracetamol, and possible application to biological samples make them ideal and inexpensive transducers for electrochemical (bio)sensors, with the potential to replace the screen-printed systems currently dominating in on-site sensors used.

**Supplementary Information:**

The online version contains supplementary material available at 10.1007/s00604-021-04792-3.

**Figure:**
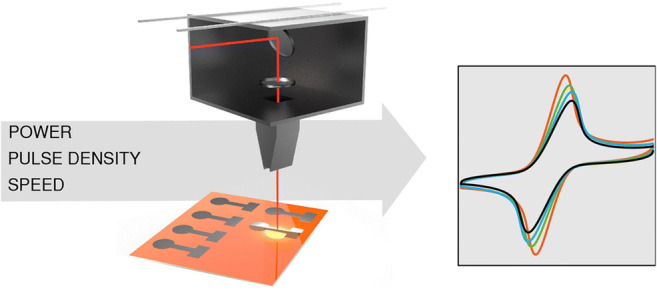
Graphical abstract

## Introduction

Low-cost, distributed electrochemical sensors can be used to address challenges in many fields of modern life, such as environmental monitoring, industrial manufacturing, and food production, and—most prominently—healthcare. Exemplary electrochemical biosensors for the measurement of blood glucose concentration have matured through decades of research and were successfully commercialized by several companies [1, 2]. These small disposable electrode strip sensors combined with a handheld glucose meter and supplied with a few microliters of blood from a pin-prick enable now millions of diabetes patients to manage their daily lives. Motivated by this success story, researchers hope to enable users to measure many other chemical parameters related to health, mostly applied to diseases where the patients benefit from continuous or frequent monitoring. While the proposed sensors naturally feature different details in their detection mechanism to provide suitable sensitivity and specificity for a given application, many rely on the same concept of electrochemical detection with a disposable, modified electrode, which means electrode materials as well as the production process need to be reliable and inexpensive. At the moment, the most common commercial electrode type is the screen-printed carbon electrode [3–5]. However, in most recent years, among the many nanomaterials proposed as better electrode material, laser-induced graphene (LIG) was described which has the potential to truly challenge and eventually substitute screen-printed electrodes (SPEs) [6, 7].

LIG is a porous graphene-like material that can be created by simply pointing a sufficiently strong CO_2_ laser onto commercial polyimide foil (e.g., Kapton®) in an ambient environment. The exact mechanism of the conversion has not been fully described and is also not the topic of this publication, but the following can be assumed to take place. A strong focused laser beam with a pulse length on the order of microseconds locally heats the substrate to temperatures above 2500 °C [8] followed by rapid cooling. At least part of the irradiated polymer immediately melts/evaporates, causing the porous nano- and microstructure observable by scanning electron microscopy (SEM). Photothermal [8] bond breaking rearranges the structure into mostly sp^2^-carbons while the carbon content increases from below 70 to above 90%, as non-carbon elements partly remain in the material, possibly incorporated as heteroatoms in the dominating hexagonal carbon rings, but mostly evaporate as gases. Carbonization is readily confirmed by a color change from orange to black. Examination of the created material via transmission electron microscopy (TEM), X-ray diffraction measurement (XRD), and Raman spectroscopy further reveal high similarity to few-layer graphene, confirming the sp^2^ carbon structures and giving rise to the name laser-induced graphene [6, 9].

With a commercial computer-controlled laser cutter, one can create two-dimensional patterns, a process generally termed direct laser writing (DLW). The obtained LIG patterns can have a spatial resolution of roughly 25 to 150 μm, depending on the employed focusing lens, and can be used as electrical transducers for various applications. DLW on Kapton film was first reported by the Tour group at Rice University in 2014 which also coined the term LIG and emphasized its possible application for the production of microsupercapacitors [6]. In subsequent publications, they reported areal capacitances of 4–16.5 mF/cm^2^ for pristine and heteroatom-doped LIG, which could be raised to 934 mF/cm^2^ by deposition of MnO_2_ for additional pseudocapacitance [10]. Since then, the material has been further characterized and variations of the manufacturing process have been explored by choosing different substrates—among them wood, paper, and even coconut shells—or by changing the composition of the lasing atmosphere [11, 12]. Much of this has been covered in two recent reviews published by the Tour group [9, 13].

Since LIG is a graphene-like pure carbon material that can be made inexpensively in any chosen two-dimensional shape, it seems an obvious candidate for disposable electrodes used in electrochemical sensing, an area, which is dominated by screen-printed carbon electrodes (SPCE) at the time. The largest existing market for disposable biosensors is blood glucose monitoring for diabetes patients and commercial electrodes used in blood glucose sensing are made by screen-printing, an inexpensive method suitable for mass production. In this method, a suspension of conductive material is transferred onto a support through a fine mesh, except in those places covered by a stencil, defining the desired shape of electrodes, leads, and contacts. Afterwards, the pattern is baked to solidify the suspension. With LIG electrodes, on the other hand, no material other than the polyimide foils is needed, and no baking step is necessary; the pattern is created without the need for any additional binder substances and can thus easily withstand most organic solvents. Furthermore, less material is wasted in this process and a change in pattern design is easily realized as it only requires a new drawing on the computer. In contrast, stencils must be prepared for each design change in the SPCE printing process. These differences suggest that LIG electrodes have a high potential to become a preferable electrochemical transducer for point-of-care applications as their production ought to be simpler, less expensive, and easily adaptable to a roll-to-roll fabrication process. We and others have employed LIG for electrochemical sensors, e.g., Nayak et al. have shown that the oxidation peaks for the biological analytes dopamine, ascorbic acid, and uric acid could be well resolved in pulsed voltammetry on LIG [14]. Fenzl et al. used pyrenebutyric acid to couple a thrombin aptamer to the LIG matrix and demonstrated that it can be successfully used in an aptasensor [15]. Also, recent work from our lab describes a combination of amperometry-, potentiometry-, and impedance-based sensors made from LIG for the measurement of lactate, potassium, and conductivity in sweat [16]. Other groups have modified DLW-created carbon electrodes with copper nanoparticles alone or in combination with diamine oxidase to detect glucose or biogenic amines respectively [17, 18]. The group of Claussen has used LIG to make ion-selective electrodes for the detection of ammonium and nitrate in soil [19] or ammonium and potassium in human urine [20]. Cardoso et al. and Beduk et al. reported the successful use of molecularly imprinted polymers (MIP) on LIG electrodes to detect chloramphenicol and bisphenol A, respectively [21, 22]. More examples for the use of this material in electrochemical biosensing are listed in recent review papers by Kurra et al. [7] and Lahcen et al. [23].

Through all of these studies, it is generally recognized that the laser power, the laser beam size at the substrate surface, the speed at which the laser beam spot progresses during writing (i.e., the scan speed, *v*), and the spatial laser pulse density (i.e., how many times the laser fires per distance traveled over the substrate) all influence the procedural outcome. That is, they give rise to different structures in the nano- and micrometer regime, as well as differences in the Raman spectrum, electrical conductivity, and hydrophilicity of the interface. We postulated, therefore, that these parameters will have a significant impact on LIG’s usability as electroanalytical transducer since electroanalytical performance is mainly determined by conductance, electron transfer, and electrode surface area. Here, we report on a detailed study investigating the performance of LIG electrodes fabricated over a large parameter space of laser power, scan speed, and different pulse densities in an effort to provide a systematic evaluation and understanding of how LIG fabrication parameters influence performance in electrochemical sensing. The reported observations were made with a specific commercial flatbed laser engraving system but can be translated to other machines that operate in a similar manner. The findings will also support future work on binder-free carbon electrode materials and help elucidate the relative importance of LIG characteristics on actual electroanalytical performance in real-world settings.

## Materials and methods

### Chemicals

Potassium ferricyanide (K_3_[Fe(CN)_6_]), potassium ferrocyanide (K_4_[Fe(CN)_6_]), hexaammineruthenium trichloride ([Ru(NH_3_)_6_]Cl_3_), dopamine hydrochloride, uric acid, L-ascorbic acid, p-nitrophenol, acetaminophen, and methylene blue were obtained from Sigma Aldrich in analytical grade. Chemicals needed for preparation of phosphate-buffered saline (PBS, 8.1 mM Na_2_HPO_4_, 1.9 mM KH_2_PO_4_, 2.7 mM KCl, 137 mM NaCl, pH 7.4) were bought from Merck (Germany) or Carl Roth (Germany) in analytical grade. All chemicals were used without further purification. Non-adhesive Kapton HN foil (125 μm) was bought from CMC Klebetechnik GmbH (Frankenthal/Pfalz, Germany), and conductive silver paint was obtained from Busch GmbH & Co. KG. (Viernheim, Germany).

### Electrode fabrication

**Fig. 1:**
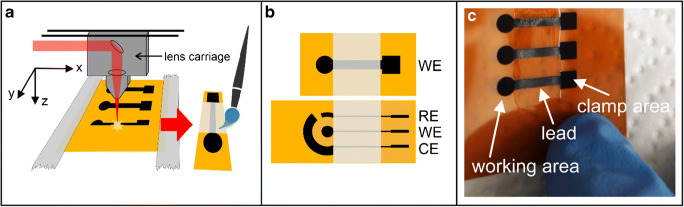
Electrode fabrication scheme (**a**), sketches of used electrode designs (WE working electrode, CE counter electrode, RE reference electrode), top = “simple” design, bottom = 3-electrode design (**b**), photo of prepared LIG electrodes (**c**)

Electrode patterns were designed on a computer with the vector graphics software CorelDraw. A model VLS2.30 laser cutter (Universal Laser Systems, Scottsdale, AZ, USA) equipped with a 30 W CO_2_-laser (10.6 μm) and focused beam diameter of approx. 125 μm (2″ lens) was used to create LIG electrodes on Kapton foil. The foil was cut into sheets of suitable size and the borders were fixed with tape directly onto the machine’s engraving table which was brought into focal distance. The *z*-distance yielding the smallest laser spot mark on a test material was regarded as the accurate focal distance with an absolute value of an estimated 5.1 ± 0.1 cm. A fabrication scheme and two examples for electrode designs are shown in Fig. 1.

The power setting (1 to 100% of 30 W), movement speed of the lens carriage in *x*-direction (1% to 100% of 50 inches/s), and spatial laser pulse density (with the fixed combinations of 500 by 500, 1000 by 1000, or 1000 by 2000 PPI (pulses per inch in *x*- and *y*-direction)) were varied using the machine software. Percent values are used here instead of physical units to report power and speed settings (e.g., “1% power” instead of “0.3 W”) because % power and measured power scale linearly only approximately so that a direct translation into physical units may in some cases be imprecise. For brevity, the following shorthand was adopted for scribing conditions, in which e.g., “1/10/1000 × 2000” means “1% power, 10 % speed, 1000 PPI in *x*-direction, and 2000 PPI in *y*-direction.” The distinction between *x*- and *y*-directions, indicated in Fig. 1a, is meaningful because the lens carrier will only travel quickly in the former and more slowly in the latter direction, which is inevitable due to the way the positioning system is constructed, as the laser moves with the set speed and frequency along the *x*-axis followed by a move in the *y*-direction to then continue the scan at the set speed and frequency back along the *x*-axis. Nonetheless, the device delivers the desired PPI in each direction. Air was extracted from the scribing chamber continuously during operation to remove soot and any emerging gases.

Pure argon and nitrogen gas could be supplied directly to the processing chamber to modulate the atmosphere. The gases were released directly at the lasing spot through the air-assist cone (see Fig. S1 in Online Resource 1). However, in contrast to the experiments with controlled-atmosphere chambers done by Li [12] and others, the supplied gases were inevitably diluted with air that was continually sucked into the (non-airtight) scribing chamber by the extraction system. Therefore, the lasing atmosphere was enriched in Ar or N_2_ but measured data on gas composition is not available.

After scribing, electrodes were rinsed with water, then isopropanol, and then dried under a nitrogen stream. Transparent nail polish was painted over the leads to restricting contact with electrolyte solution only to the electrode area. Post-scribing steps can be viewed in the supplied video files (Online Resources 2, 3, 4, 5). Electrodes were stored in non-airtight boxes at room temperature in the lab, until used.

### Electrochemical analysis

For all electrochemical measurements, the analyte was dissolved in 1 × PBS (pH 7.4). A PalmSens4 potentiostat (PalmSens BV, Netherlands) was used. For screening measurements, in which the same electrolyte could be reused many times, the simpler electrode design version of LIG (Fig. 1b, top) was clamped as working electrode (WE) and dipped into approx. 10 mL of electrolyte together with a platinum wire as a counterelectrode (CE) and a pole Ag/AgCl reference electrode (RE) (BAS Inc., USA). On the other hand, the 3-electrode design (Fig. 1b, bottom) was more convenient for the recording of calibration plots and working with small volumes of electrolytes. Here, a 50-μL droplet was placed onto the LIG electrode (WE and CE) and a Ag/AgCl reference electrode was contacted from above. The small LIG electrode in the 3-electrode design was intended as the basis for a future reference electrode but not used in these investigations. Figure S2 shows photos of both cell arrangements. Voltammetric peak potentials and heights were detected either fully- or semi-automatically through the software PSTrace 5.8 with a linear baseline. For the fabrication parameter survey, cyclic voltammetry (CV) of 5 mM K_3_[Fe(CN)_6_] in PBS + 0.1 M KCl (pH 7.4) was carried out with a scan rate of 50 mV/s at 1 mV steps and the peak-to-peak distance was calculated as Δ*E*_p_ = *E*_p,ox_ − *E*_p,red_ to serve as a measure of electrode quality. CV with scan rates between 25 and 200 mV s^−1^ was then recorded for a selected electrode type to determine its electrochemically active surface area (ESA) and effective heterogeneous electron transfer rate for (*k*^0,eff^), as described in the supplementary information. Square-wave voltammetry was used for quantitative analysis and run at 5 mV step, 50 mV amplitude, and a frequency of 10 Hz.

### Sheet resistance

The electrical sheet resistance was measured with a four-point-probe apparatus built in-house: four spring-loaded golden pins, arranged in a straight line and spaced equally by 1.5 mm were used to contact the center of a 1- × 1-cm LIG sample. The probing current was 100 μA.

### Physical characterization

Infrared reflectance spectra were recorded over the range of 4000 to 650 cm^−1^ (8 cm^−1^ resolution) on an Agilent Cary630 equipped with a diamond single bounce attenuated-total-reflectance attachment. 32 scans were averaged for sample and background each. The elemental composition of Kapton and LIG was measured via combustion analysis on a Vario Micro Cube. Raman spectra were collected from 50 to 3500 cm^−1^ on a Thermo Fisher DXR Raman microscope with a 532-nm laser set to 8-mW power and a ×50 objective with an estimated focal spot diameter of 0.7 μm. 16 scans were averaged per spot.

X-ray photoelectron spectroscopy (XPS) studies were carried out in a Kratos Axis Supra DLD spectrometer equipped with a monochromatic Al Kα X-ray source (*hν* = 1486.6 eV) operating at 150 W and a vacuum of ~10^−9^ mbar. Survey and high-resolution spectra were collected with pass energies of 160 eV and 20 eV respectively. Samples were mounted in floating mode in order to avoid differential charging. Charge neutralization was required for all samples. Binding energies were referenced to the sp2 hybridized (C=C) carbon for the C 1 s peak set at 284.4 eV from graphene.

SEM images were obtained with a Zeiss LEO 1530. Water contact angles were recorded with the sessile drop method on an OCA 25 system with corresponding software (DataPhysics Instruments GmbH, Filderstadt, Germany).

## Results and discussion

Laser-induced graphene was studied as an alternative, carbonaceous material for electrochemical (bio)sensing applications. Its promising characteristics described by us earlier [15, 16] indicate that it may be not only an alternative but a superior transducer material for electrochemical point-of-care sensors. Not much is known about the effect of energy input during the fabrication in correlation to electroanalytical performance, only anecdotal data are available describing obtainable micromorphologies. By systematically studying process parameters that influence the energy input and correlating it to micromorphologies, Raman, and especially electroanalytical characteristics, this knowledge gap was sought to be filled by the study.

### Understanding of scribing parameters’ effects on laser-induced graphene

We created LIG electrodes with a circular working area (*d* = 3 mm, *A* = 0.071 cm^2^) connected via a bridge to an electrical contact area for electrochemical testing (see Fig. 1c). To assess the influence of available laser instrument parameters, the power- and speed settings as well as spatial pulse density were systematically varied.

**Fig. 2:**
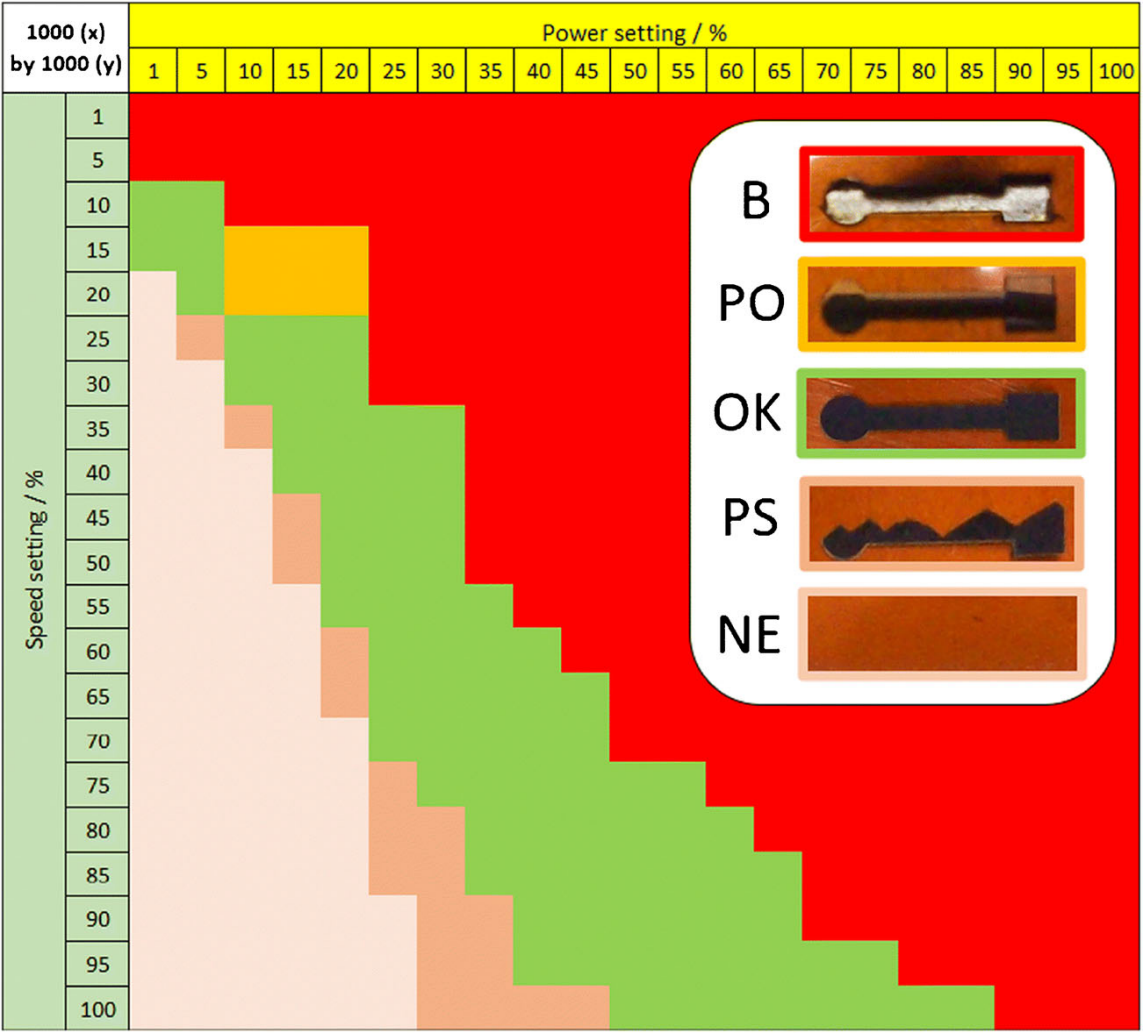
Heatmap of electrode outcome vs. power and speed settings at a pulse density of 1000 × 1000 (*x* by *y*) (color code as indicated in the inset: green = ok, darker brown = partial scribing (PS), lighter brown = no effect (NE), orange = LIG peeled off from substrate (PO), red = laser burned through substrate (B))

The electrodes were judged by mechanical integrity and visual appearance, represented by the heatmap overview for a medium pulse density setting (Fig. 2). Representative different visual appearances are shown in the inset. Electrodes of homogenous texture that would withstand bending without delamination were created by a suitable combination of power and speed (area of green color in Fig. 2). On the contrary, too much energy input resulted in brittle electrodes that would peel off the substrate easily. This was the case when the power setting was not ideal and hence a bit too high or the speed a bit too low (red and orange color). At low power and high-speed settings, the substrate was not carbonized or only partially (brown colors). When the energy input was just at the lower limit necessary for carbonization, only part of the desired shape was carbonized and instead triangular shapes appeared (see the second electrode from bottom in the inset of Fig. 2). This odd phenomenon stems from the carbonization starting at randomly located but energetically favorable nucleation points on the substrate. Those initially carbonized islands then promote carbonization in the immediate vicinity through increased absorbance which causes the lines of converted LIG to become longer with each consecutive sweep and create the observed triangular patterns with the tip facing upwards. The tips face downwards when the laser scanning direction is reversed (i.e., going from bottom to top regarding the *y*-direction).

**Fig. 3:**
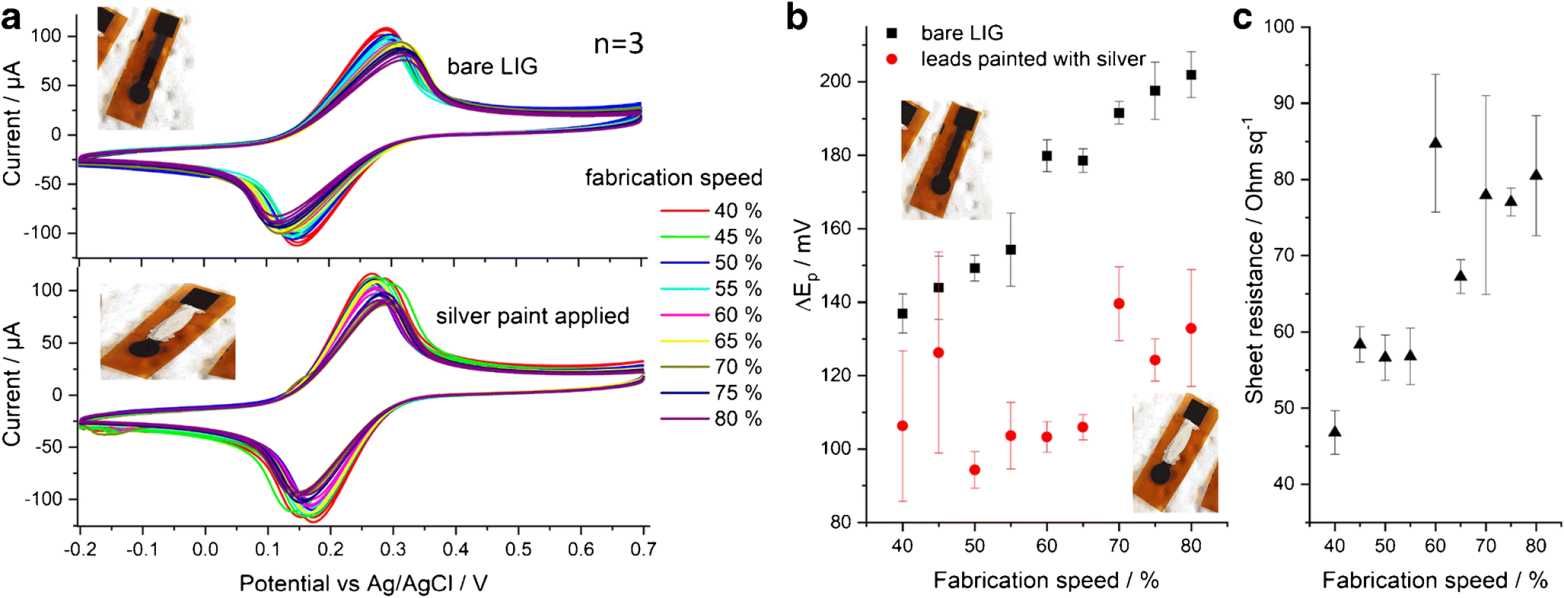
**a** CV of 5 mM K_3_[Fe(CN)_6_] in PBS with electrode leads bare or covered with silver paint. Higher speed setting correlated with larger Δ*E*_p_ (laser power = 30%, 1000 × 1000 PPI). **b** Peak-to-peak separation in CV. **c** sheet resistance of LIG vs scribing speed

While all green-marked laser settings generated visually similar electrodes, conductivity, and electrochemical activity differed. Cyclic voltammograms (CVs) were recorded on a series of electrodes in whose fabrication all parameters but one were kept constant to demonstrate the influence of a single parameter. The settings of 1000 × 1000 PPI and a constant power of 30% were chosen, because at this point, the speed could be varied on a wide range while producting functional electrodes (see Fig. 2). The resulting CVs in Fig. 3a show that peak-to-peak separation (Δ*E*_p_, Fig. 3b) and sheet resistance (Fig. 3c) both increase with scribing speed. To remove the influence of lead resistance from CV analysis, the leads were painted with conductive silver paste. Consequently, Δ*E*_p_ values dropped overall and were influenced less by the scribing speed. Apparently, the high resistance of the LIG leads causes significant potential drop (iR-drop) between electrode working area and connecting clamp, which distorts the shape of the voltammograms. Longer leads caused higher drop (resistivity factor) but also larger electrode surfaces, higher concentration of redox species, or increased scanning speed in potential sweeping experiments (current factor). Strategies to prevent iR-drop therefore include the use of small electrodes and low redox species concentrations, if application of conductive paint is to be avoided. The lead dimensions of a designed electrode are usually dictated by practical reasons and therefore offer little room for adjustment. However, with optimized laser settings (see below), the sheet resistance of LIG could be reduced to as low as 10–20 Ω sq.^−1^ which greatly reduced the iR-drop problem (the value of 10–20 Ω sq.^−1^ was not a specific target, but it was the best achievable). Therefore, it was possible to make an all-LIG electrode without additional processing steps, like painting the leads, when the right scribing conditions are used.

Furthermore, the influence of laser pulse density was investigated. The laser cutter used in this study permitted only certain locked pulse density settings. Hence, 500 × 500 PPI, 1000 × 1000 PPI, and 1000 × 2000 PPI were compared (*x-* by *y*-direction). The heatmap in Fig. 2 is collected at 1000 × 1000 PPI which resulted in a particularly good set of electrodes at a broader range of settings. Some power/speed combinations at the higher pulse density of 1000 × 2000 PPI yielded even better conductivity and probably electron transfer. However, these electrodes were sometimes prone to delamination and the design-space for power and speed was narrower at the increased pulse density (see Fig. S3). Therefore, the medium setting of 1000 × 1000 PPI was used in this work to create LIG electrodes for sensing applications. Furthermore, in terms of output, scribing the same pattern at 2000 PPI in *y*-direction takes twice as long compared to 1000 PPI. Power/speed heatmaps recorded at other pulse density settings can be found in Fig. S3 along with a comparison of CV performance among the best electrodes obtained at different pulse density settings in Fig. S4. Electrodes created with the lowest spatial pulse density option of 500 × 500 PPI ranked lowest in conductivity and were deemed less useful for electrochemical measurements.

It should be mentioned that the exact results of Fig. 2 (and the other maps) are likely not directly translatable to machines with a different maximum laser power or different size of focused beam. Even the size of the desired pattern or placing the substrate too close to the limit of the scribing area can influence the carbonization outcome. However, from experience with different flatbed laser systems, we have found that optimal power/speed conditions always follow more or less the green diagonal region in Fig. 2. Specifically, as a rule of thumb, it seems that low power, low speed, and high spatial pulse density will create LIG electrodes with high conductivity and generally good electron transfer behavior at the electrode-electrolyte interface.

Based on these findings, the setting of 1% power, 10% speed, and 1000 × 1000 PPI (1/10/1000 × 1000) was selected as a very conductive, electrochemically active, and mechanically sturdy LIG material. Unless different settings are specifically mentioned, these settings were used to produce electrodes for all following electrochemical tests, and scribing took about 1 min per electrode. It should be pointed out though that many of the settings within the green range of the heat map (Fig. 2) can be suitable for a user’s electroanalytical needs and be created at higher throughput.

**Fig. 4:**
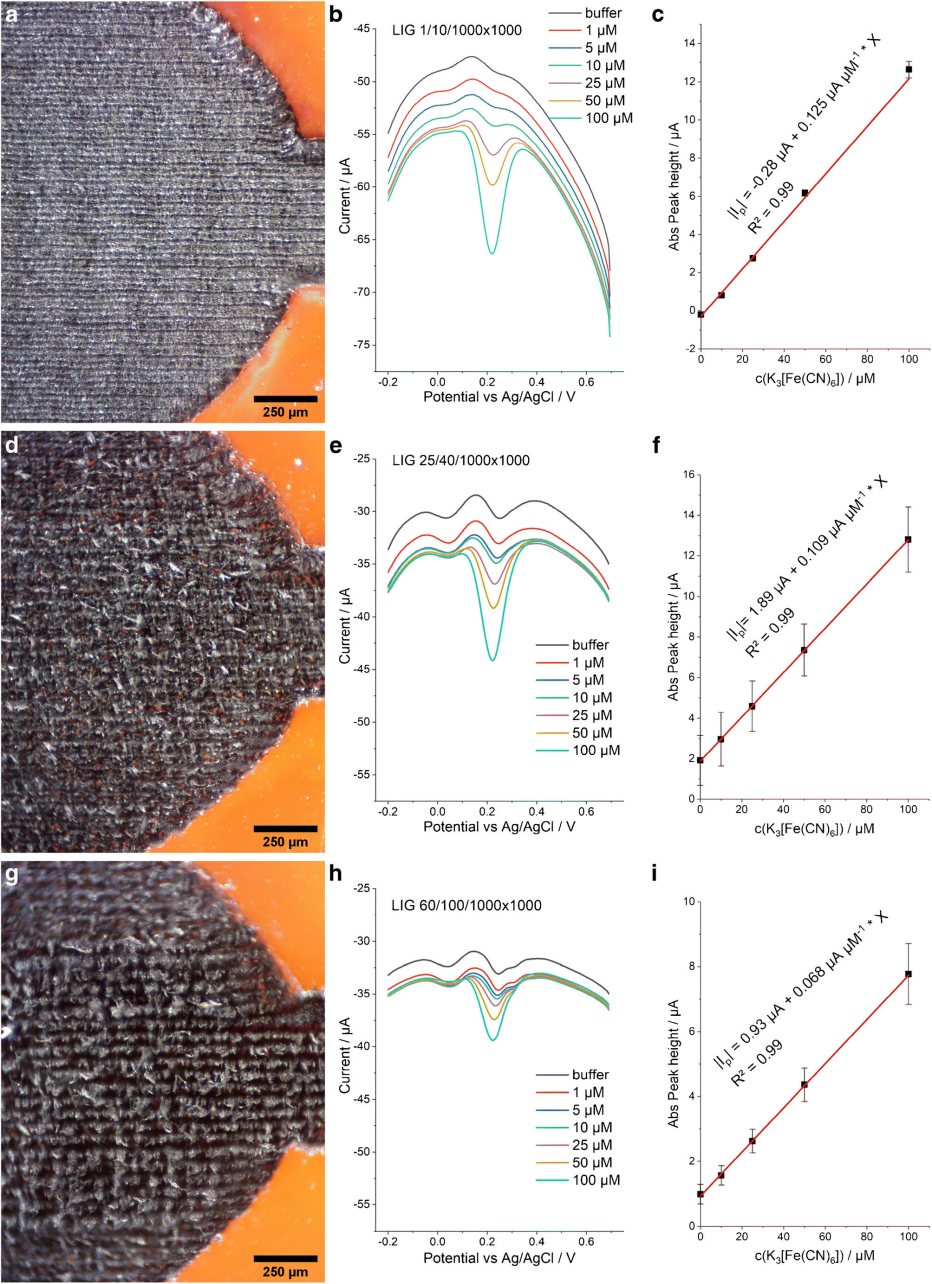
Micrographs showing the active region of LIG electrodes made with the same pulse density but different power and speed settings (**a**, **d**, **g**). Square-wave voltammograms (**b**, **e**, **h**) and dose-response plots (**c**, **f**, **i**) of ferricyanide standards on those electrodes. *E*_begin_ = 0.7 V, *E*_end_ = −0.2 V, step = 5 mV, pulse = 50 mV, *f* = 10 Hz. All standards (V = 50 μL) were measured on the same electrode in order of rising concentration. 3-electrode design LIG electrodes were used

To demonstrate the variation in chemical sensing performance within the same pulse density setting, three electrode types made with increasing energy input were chosen from the green area in Fig. 2: 1/10, 25/40, and 60/100 (the first number denotes %power, the second %speed), and SWV responses were recorded to standards of [Fe(CN)_6_]^3−^ in concentrations between 1 and 100 μM (Fig. 4). The sensitivity (see Fig. S16) decreases from type 1/10 over 25/40 to 60/100 which correlates with rising sheet resistance of the different LIG types: (26 ± 0.6) Ω, (49 ± 1.5) Ω, (57 ± 6) Ω for 1/10, 25/40, and 60/100, respectively. While all three electrode types appeared visually homogenous, the microstructure of 1/10 appears most uniform and flat while 25/40 shows fibrous structures of LIG and gaps. This appearance is even more pronounced in LIG type 60/100.

**Fig. 5:**
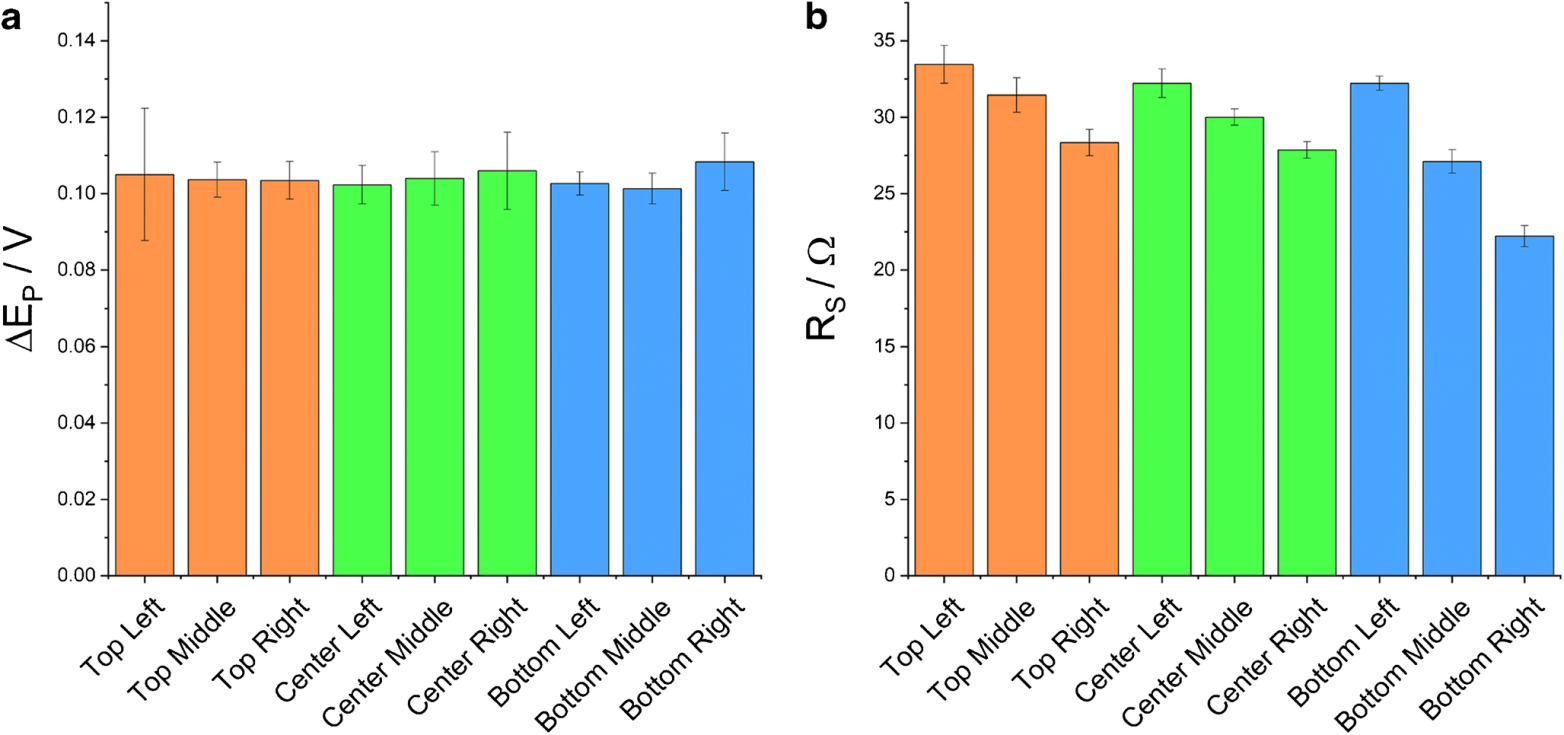
Peak-to-peak distance obtained with LIG electrodes in CV (**a**) and sheet resistance of LIG samples (**b**) (*n* = 3)

One concern about electrode manufacturing reproducibility regarded the location of the polyimide substrate in the machine when being scribed, since the lens carrier might be slower in some regions than in others due to available acceleration distance. Electrodes were thus scribed in several relative locations on the engraving table and peak-to-peak distances were measured. Also, 1- × 1-cm squares of LIG were made in the same locations to measure the sheet resistance. It was found that substrate location had no significant impact on performance in CV (Fig. 5a), measured as peak-to-peak separation. All electrodes showed around 100-mV peak-to-peak separation but the—overall low—sheet resistance values dropped from 33 Ω at the top left location to 23 Ω in the bottom right position (Fig. 5b). We conclude that while the effect of location on sheet resistance may be kept in mind, for most electrochemical experiments, no special attention needs to be paid as to where the substrate is placed during electrode manufacture.

It is known that the presence of doping atoms such as nitrogen can increase the overall conductance of graphene-like materials [24]. It was therefore investigated if such doping could be achieved by simply changing the gas environment during the scribing process. Therefore, the laser cutter was flooded through a port in the chamber with either nitrogen or argon during operation. While this simple approach admittedly did not create a perfect gas atmosphere, it can quickly be realized in any lab. The LIG surfaces prepared under argon and nitrogen became very hydrophobic with water contact angles of 170° and 150° respectively, while the sessile drop spread completely on LIG prepared under ambient atmosphere. The gas environment influenced the microstructure (Fig. S9) and also the Raman spectrum (Fig. S10) but oddly seemed not to cause significantly different outcomes in cyclic voltammetry (Fig. S11). In terms of large-scale fabrication, this indicates that the ambient atmosphere is sufficient to produce high-quality electrodes. More in-depth investigations regarding the influence of lasing atmosphere on spectral characteristics and hydrophobicity of LIG were published by Li et al. and Mamleyev et al. [12, 25]

### Physicochemical characterization

Infrared reflectance spectra of the Kapton substrate and LIG are displayed in Fig. S5A. The features of polyimide in the fingerprint region between 600 and 1800 cm^−1^ completely disappeared and the overall transmission dropped profoundly across the whole spectrum after carbonization. Both observations confirm the chemical transformation to carbonaceous LIG.

The carbon content increased from roughly 68% in Kapton to above 93% in LIG (Fig. S5B), indicative of carbonization, while contents of hydrogen and nitrogen decreased from 3 and 7% to values below 1%, likely being released as gases during the scribing process. The oxygen content dropped to 5% after scribing, which points toward the presence of oxygen-containing groups in the carbon lattice of LIG, which were also found via XPS analysis (Fig. S7). The Raman spectrum of LIG features the characteristic *D*, *G*, and 2D peaks known from graphene-like materials (Fig. S6).

**Fig. 6:**
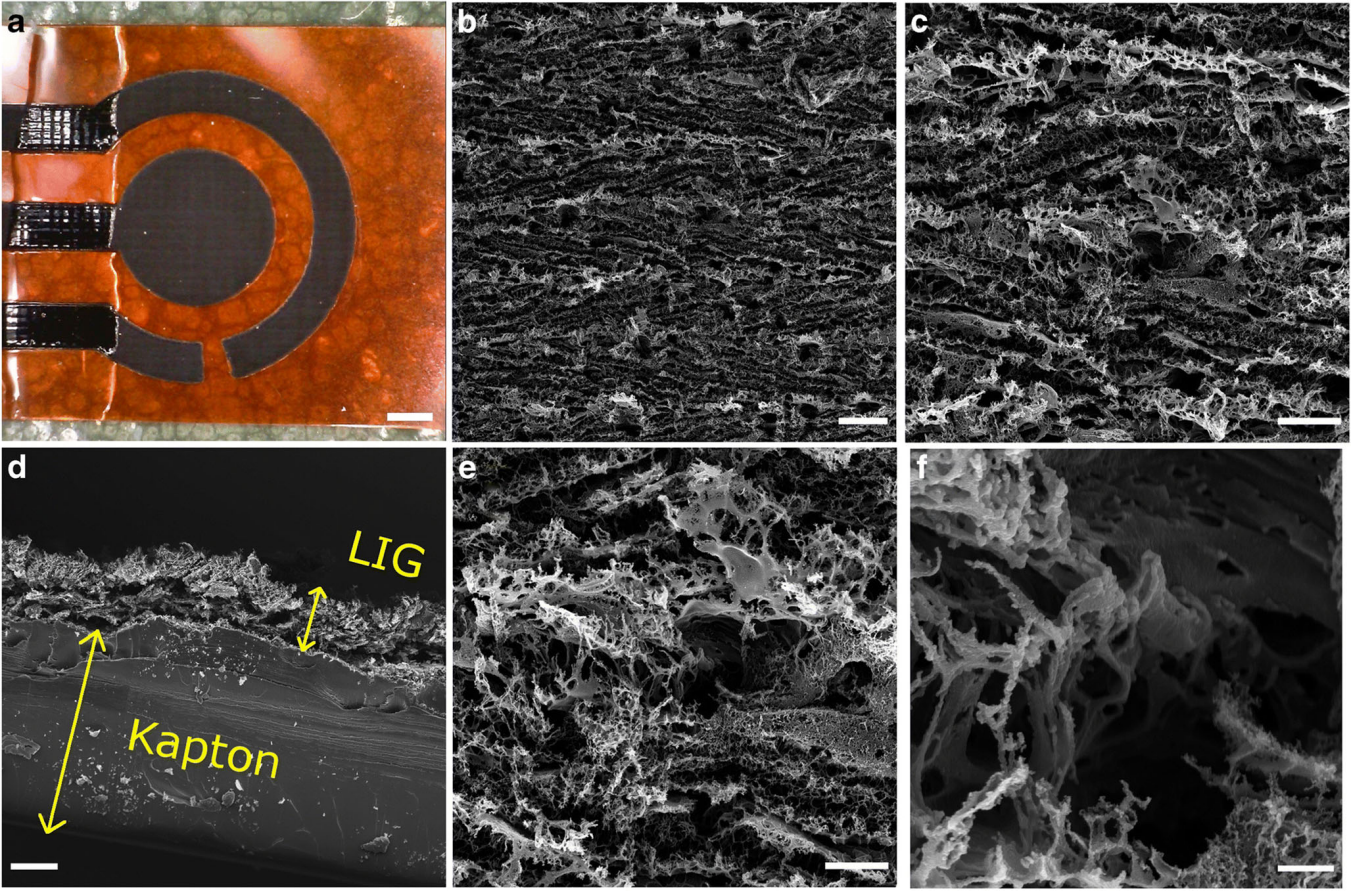
Photograph and SEM micrographs of LIG (1/10/1000 × 1000) scale bars in pictures **a**–**f** are 500, 20, 10, 20, 5, and 1 μm

SEM micrographs of porous LIG of the type 1/10/1000 × 1000 are shown in Fig. 6. A pattern of horizontal trenches is visible at low magnification (Fig. 6b), which was created when the pulsed laser beam passed over the substrate in successive lines from top to bottom with a pitch of about 25 μm. Given this pitch and the beam diameter of approximately 125 μm, each location on the surface specified by the pattern was irradiated an average number of 18 times because of laser beam overlap in *x*- and *y*-directions (see also Fig. S8).

As seen in the cross-sectional view in Fig. 6d, a significant part of the Kapton substrate, around 100 μm, remains intact after lasing and serves as a support for the more delicate coral-reef-like LIG structure which has an average height of 27 ± 3 μm as determined by SEM.

SEM pictures of LIG obtained at other scribing conditions revealed significantly different morphologies. This observation has already been disclosed by others, e.g., Tiliakos et al. identified five different morphic groups of LIG with differences in Raman spectra, electrical conductivity, and wettability [8]. They used a galvanometric laser processing unit which permitted a high degree of variation in scan rate and pulse frequency. On the other hand, Duy et al. reported the creation of long LIG-fibers (LIGF), by decreasing the pulse frequency while at the same time using a smaller beam diameter, effectively reducing the beam overlap [26]. The commercial flatbed laser processing unit used by their group is similar to the one in this study and we obtained LIGF by reducing pulse densities to 500 PPI but still using a beam diameter of 125 μm (Fig. S12). Apparently, the fibrous structure was also created when a certain beam overlap occurs, in this case approx. 4 times. In fact, even at 1000 × 1000 PPI, brush-like LIG structures could be observed, given the right power/speed combination, as seen in Fig. 4f above. Correlating the morphology to the electroanalytical performance in Fig. 4e, it can be deduced that large brush-like structures are not favorable for electroanalysis as likely looser structures lead to a higher resistance and hence worse electroanalytical performance. A possible gain in overall surface area hence does not translate here into better electrodes for analysis.

**Fig. 7:**
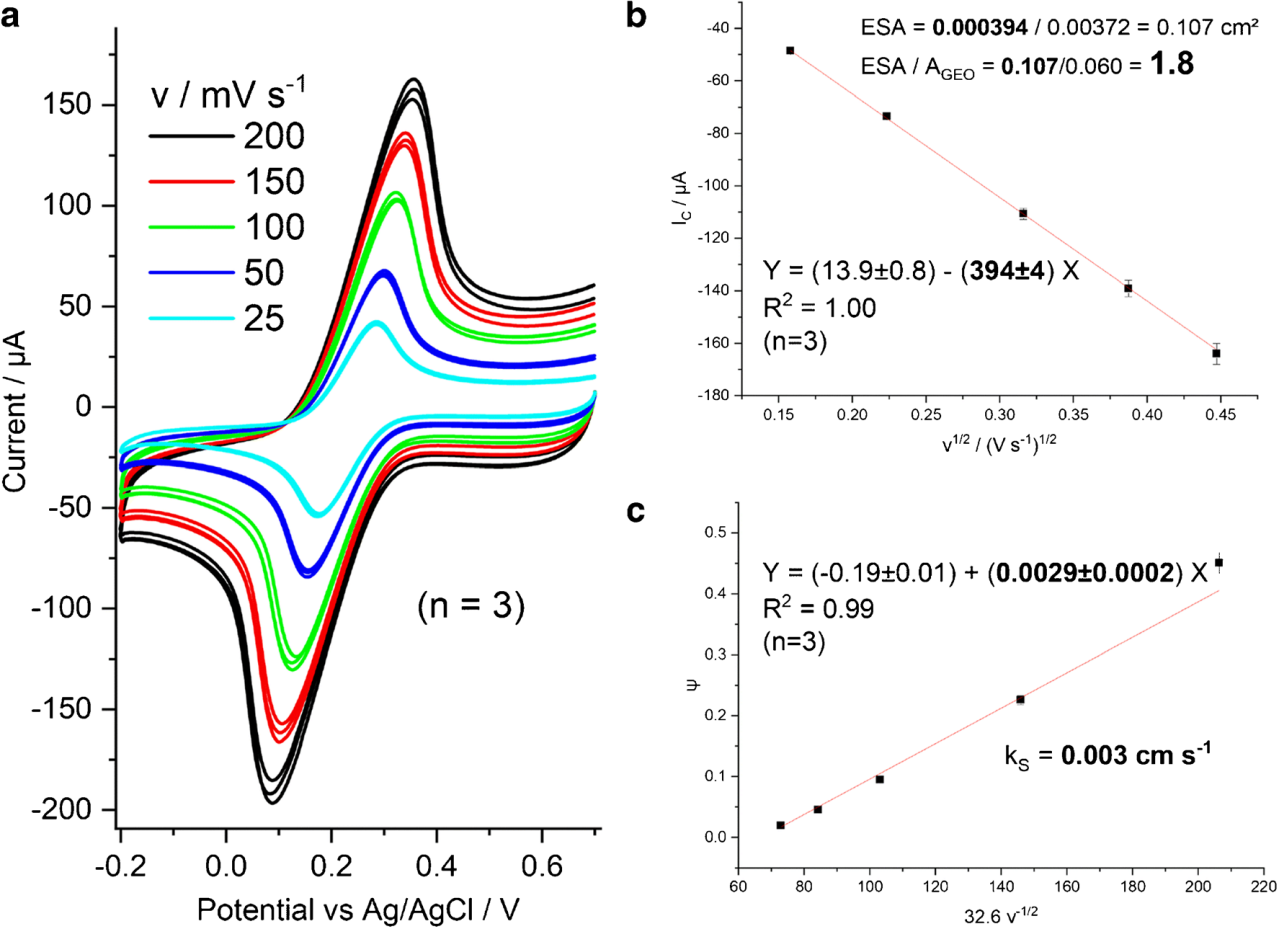
**a** CV in 5 mM K_3_[Fe(CN)_6_] in 0.1 M KCl on 1/10/1000 × 1000 electrodes at various scan rates. **b** Plot of cathodic peak current vs *v*^1/2^. The slope was divided by the factors in the Randles-Sevcik equation (0.4463 F^3/2^ (*D*/*R T*)^1/2^ C), with *F* = 96,485 A s mol^−1^, *D* = 7.63E−6 cm^2^ s^−1^, *R* = 8.314 J K^−1^ mol^−1^, *T* = 296.15 K, *C* = 5E−6 mol cm^−3^, to yield 0.107 cm^2^. Division by the geometric surface of 0.06 cm^2^ yielded a ratio of 1.8. **c** Nicholson plot based on the same data: slope = *k*^0,eff^ = 0.003 cm s^−1^. 3-electrode design LIG electrodes were used

The electrochemically active surface area (ESA) of LIG electrodes, determined with the voltammetric method via the Randles-Sevcik equation, was 0.107 cm^2^ (about 1.8 times the geometrical surface area *A*_GEO_, Fig. 7b) and the calculated effective heterogeneous electron transfer coefficient (*k*^0,eff^) for the [Fe(CN)_6_]^3−/4−^ couple was 0.003 cm s^−1^. For comparison, commercial screen-printed carbon electrodes exhibited a lower calculated ESA of 0.9 times *A*_GEO_ and a comparable *k*^0,eff^ of 0.002 cm s^−1^. However, the Randles-Sevcik relationship—like the Nicholson method for the determination of *k*^0,eff^—is only strictly applicable to smooth electrode surfaces with planar semi-infinite diffusion of dissolved redox species. Therefore, the values of ESA and *k*^0,eff^ reported here should be regarded as an estimate rather than accurate. The specific surface area of LIG 1/10/1000 × 1000 determined by nitrogen adsorption isotherm analysis was approx. 330 times the geometrical surface area. Obviously, this does not correlate to the determined ESA. We assume that the electrochemical experiment solution may not enter all of the pores and cavities due to hydrophobic pouches and that some areas are not conductively connected well enough, which leads to this dramatic difference in measurements.

Table 1 compares the values of ESA, *k*^0,eff^, and sheet resistance to previously published data for LIG or very similar material (LSG). The values we report here are in the spectrum of previously reported results.

**Table 1:** Electrode material characteristics compared to previous publications of similar materials (*NA*, not available; ESA and electron transfer rate coefficient were in all cases determined with the voltammetric method)

Laser	Substrate	*A*_GEO_ (cm^2^)	Roughness factor (ESA/A_GEO_)	Sheet resistance (Ω sq^−1^)	*k*^0,eff^ (cm s^−1^)	Ref.
788 nm diode	Graphene oxide	0.16	NA	80	0.0133	[27]
788 nm diode	Graphene oxide	0.07	NA	589	0.0237	[28]
10.6 μm, CO_2_	Kapton	0.07	1.8	NA	0.0044	[15]
405 nm diode	Kapton	0.13	2.7	NA	0.0030	[18]
10.6 μm, CO_2_	Kapton	0.07	1.5	12,700	0.0146	[29]
10.6 μm, CO_2_	Lignin film	0.07	1.8	2.8	0.0090	[30]
10.6 μm, CO_2_	Kapton	0.06	1.8	23	0.0030	This work

Finally, electrochemical impedance spectroscopy revealed extremely low impedance values compared to commercial screen-printed carbon electrodes of the same geometrical size (Fig. S13) We conceive that especially this characteristic will make LIG an interesting material for EIS sensors as also demonstrated by us and other groups previously [29, 31, 32].

### Voltammetric applications of LIG electrodes

The electrochemical behavior of various molecules of interest on LIG electrodes was investigated by CV to test the broad applicability of this electrode type for chemical sensing (Fig. S15). We observed that [Ru(NH_3_)_6_]^3+^, a representative for molecules that undergo outer-sphere electron transfer, was just as easily detected as [Fe(CN)_6_]^3−^, which is classified as a more surface-dependent redox species (Fig. S15A) [33]. The detection of dopamine (DA) can generally be hindered by the presence of ascorbic acid (AA) or uric acid (UA) which oxidize at very similar potentials. In Fig. S15B, the peaks of DA, AA, and UA are sufficiently separated on LIG as also shown by our collaborators earlier [14]. This beneficial effect might be partly explained by the transition from a planar semi-infinite diffusion regime to thin-layer diffusion, as Compton’s group has pointed out in the past about the modification of glassy carbon electrodes with nanomaterials [34]. Figure S15C and D demonstrate the detection of p-nitrophenol and paracetamol, which could be direct analytes of interest, while a CV of the common redox mediator methylene blue (MB) is shown in Fig. S15E. MB adsorbs easily onto LIG, as indicated by the very low peak separation and increased currents in consecutive scans and, in fact, Rathinam et al. already suggested LIG powder as adsorbent for MB in water treatment [35]. Since MB is also used as an electron mediator in biosensors, the strong adsorption may be beneficial in that case.

Generally, an overall high background capacitance due to the large inherent surface area can be observed on LIG electrodes, paired with an excellent ability for oxidation and reduction reactions of inner and outer-sphere electroactive species. We observed that the current response of different redox species was significantly larger on LIG compared to commercial screen-printed carbon or glassy carbon electrodes. This may be caused by not only the porous nature of LIG but also the apparent presence of many reactive edge sites (see the large *D* peak in the Raman spectrum of Fig. S6) which may cause this improved electrocatalytic activity [36]. This demonstrates the overall utility of LIG as sensitive electrode material for analytical applications.

**Fig. 8:**
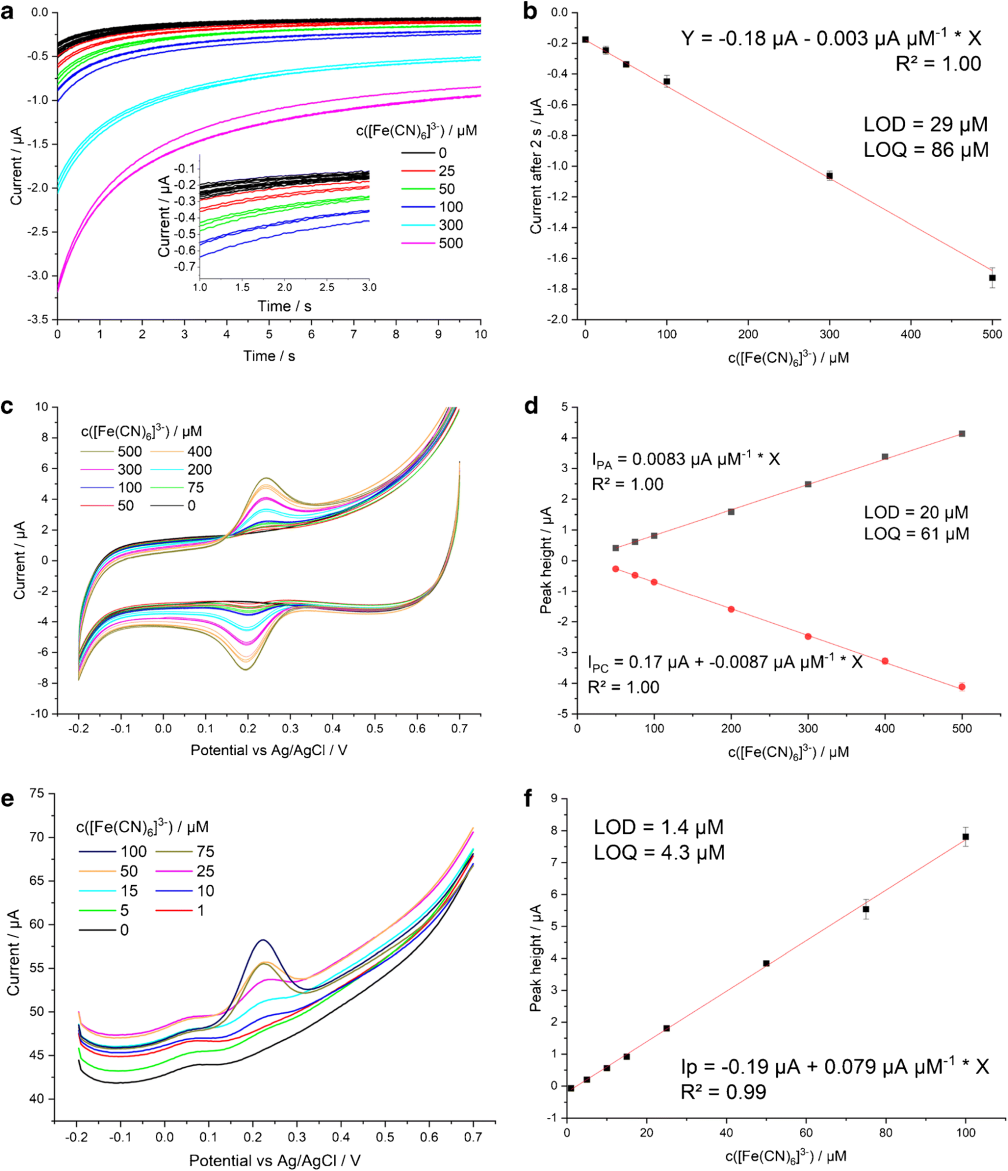
Dose-response experiments to [Fe(CN)_6_]^3−^ on LIG electrodes (1/10/1000 × 1000) using chronoamperometry (**a** + **b**, step from 0.4 V to 0.0 V), cyclic voltammetry (**c** + **d**), and square-wave voltammetry (**e** + **f**). Each electrode was used for only a single measurement and three electrodes were used per concentration level. In SWV, each fresh electrode displayed a slightly different background current and so, for clarity, each concentration level in **e** is represented by one voltammetric curve only. 3-electrode design LIG electrodes were used

Finally, we compared the detection of [Fe(CN)_6_]^3−^ on the chosen LIG electrode type using chronoamperometry (CA), cyclic voltammetry (CV), and square-wave voltammetry (SWV). Detection via CV below a concentration of 50 μM was not possible (Fig. 8c, d), the lowest detectable concentrations with CA and SWV were 25 μM and 5 μM respectively. Upwards, the response was linear to the highest tested concentration of 500 μM with CA and CV, while SWV allowed a linear calibration up to 100 μM with signals increasing less at higher concentrations (only linear part of calibration displayed in Fig. 8f).

The SWV calibration of K_3_[Fe(CN)_6_] on LIG electrodes in Fig. 8e, f can be compared to data from commercially available screen-printed carbon electrodes (Dropsens, DRP-110) in Fig. S14. Our LIG electrodes express a similar limit of detection (LOD and LOQ with SPE = 1.0 and 3.0 μM) although the screen-printed electrodes show less background current.

## Conclusion

Laser-induced graphene (LIG) was investigated with focus on understanding the effect fabrication parameters and morphologies have on the electroanalytical performance. It was found that the material properties can be tuned in the production process, and, in fact, a large range of workable parameters was found, which provides good leverage for large-scale, maximum productivity at sufficient electrode performance, tailored toward the desired application. The best analytical electrodes were obtained at low, frequent energy input, i.e., low laser power, low scanning speed, and high spatial pulse density. Key features of the material are its large porosity, sufficient mechanical stability, and chemical purity as no binder substances are required such as in screen-printed graphite electrodes. Thorough electroanalytical characterization and application to a variety of analyte molecules reveal LIG to serve well for detection in the low micromolar range and provide reliable data. As to be expected for a crystalline carbon material, some chemical species irreversibly and readily adsorbed onto LIG, which suggests its strength for single-use electrode systems. This works well with most healthcare applications, where the single-use approach dominates the diagnostic market. The nature of the laser patterning process poses two main constraints on LIG: the features cannot be much smaller than 100 μm and the resulting material is always porous and never flat. Although recently, a group reported LIG with a slightly higher resolution of 50 μm by switching the IR-laser for UV [37], a much lower feature size on the scale of a few μm or even nanometers, as achieved by lithography, is generally impossible for LIG. It is therefore not suited to create nanoscale interdigitated arrays, e.g., for detection strategies using redox cycling [38]. However, the resolution is comparable to screen-printing and LIG is very well suitable for general use [39].

A perceivable drawback of the binder-free LIG electrode material is its lower mechanical stability. For example, the highest conductive LIG cannot be used as an electrode as it lifts off its substrate when slightly bent. It has been proposed to render LIG mechanically more robust by infusion of silicon [40, 41] or cement [42], but electrode surface area and superior electron transfer processes suffer from this. Thus, instead, it is proposed that a balance between optimal conductivity and mechanical robustness needs to be found and tailored toward the final application.

## Supplementary Information

ESM 1 (DOCX 11196 kb)

ESM 2

ESM 3

ESM 4

ESM 5
