# Correction to: Process-property correlations in laser-induced graphene electrodes for electrochemical sensing

**DOI:** 10.1007/s00604-021-04904-z

**Published:** 2021-07-10

**Authors:** Arne Behrent, Christian Griesche, Paul Sippel, Antje J. Baeumner

**Affiliations:** Institute of Analytical Chemistry, Chemo- and Biosensors, University of Regensburg, Regensburg, Germany

**Microchimica Acta (2021) 188:159**

10.1007/s00604-021-04792-3

The original version of this paper was published with error in the supplementary information files. At least four of the supplementary video files (Supplementary Information 2, 3, 4, 5) were missing from the originally provided package but mentioned in article text. Given in this article are the complete list supplementary files.

The original article has been corrected.

## Supplementary Information

ESM 1 (DOCX 10.9 Mb)

ESM 2 (MP4 2107   kb)

ESM 3 (MP4 3756   kb)

ESM 4 (MP4 3667   kb)

ESM 5 (MP4 2779   kb)

