# Supplementary material for: Process-property correlations in laser-induced graphene electrodes for electrochemical sensing
Source: Mikrochim Acta. 2021 Apr 7;188(5):159. doi: 10.1007/s00604-021-04792-3 (PMC8026455; doi:10.1007/s00604-021-04792-3)
Supplement: Supplementary file 1 — (DOCX 11196 kb) [file 604_2021_4792_MOESM1_ESM.docx]

**Electronic Supplementary Material**

**Process-property correlations in laser-induced graphene electrodes for electrochemical sensing**

**Arne Behrent^1^, Christian Griesche^1^, Paul Sippel^1^, Antje J. Baeumner^1^***

1. Institute of Analytical Chemistry, Chemo- and Biosensors University of Regensburg, Germany

**Corresponding author:** Antje J. Baeumner, antje.baeumner@ur.de

Determination of the electrochemically active surface area (ESA) via cyclic voltammetry

Cyclic voltammograms of 5 mM K_3_[Fe(CN)_6_] in 0.1 M KCl (50 µL droplet placed on electrode) were recorded at different scan rates in this order 25, 50, 100, 150 and 200 mV s-1, scanning from 0.7 V to -0.2 V and back to 0.7 V (vs Ag/AgCl). After each scan, the 50 µL droplet of testing solution was replaced with a fresh one to restore starting conditions (instead of mixing, as one would in an electrochemical cell of larger volume).

For a reversible redox species, the Randles-Sevcik equation (1)(*Electrochemical Methods*, 2nd Ed. **2001**, Bard & Faulkner, chapter 6.2: Potential Sweep Methods, p. 231) describes the height of the peak in the forward scan of a CV as

$$i_{p}=0.4463\cdot n^{\frac{3}{2}}\cdot F^{\frac{3}{2}}\cdot\left( \frac{D}{RT} \right)^{\frac{1}{2}}\cdot C\cdot A\cdot v^{\frac{1}{2}} (1)$$

with

$n$ = number transferred electrons per reaction (here: 1)

$F$ = Faraday’s constant = 96485 A s mol^-1^

$D$ = Diffusion coefficient of the reactant in the electrolyte (here: 7.63 x 10^-6^ cm² s^-1^, “Handbook of Electrochemistry” 1^st^ Ed. 2007, C.G. Zoski)

$R$ = universal gas constant = 8.314 J mol^-1^ K^-1^

$T$ = absolute temperature (here: 296.15 K)

$C$ = concentration of the reactant in the bulk (here: 5 x 10^-3^ mol L^-1^)

$A$ = the electrochemically active surface area

$v^{\frac{1}{2}}$ = square root of the scan rate

The slope of the linear regression for $i_{p}$ against $v^{\frac{1}{2}}$ is the red marked term in the Randles-Sevcik equation above. Division of the slope value by all red marked symbols, except for $A$, will yield $A$, which is the ESA.

Determination of the effective heterogeneous electron transfer rate constant (k^0,eff^) via cyclic voltammetry

k^0,eff^ was calculated from CV data according to the method described by Lavagnini et al. (Electroanalysis, **2004**, 16(6), 505-506) building on the work of Nicholson (Anal.Chem., **1965**, 37, 1351) and Klingler and Kochi (J. Phys. Chem., **1981**, 85, 1731) . The method is reproduced briefly below. For our calculations, we adopted the nomenclature k^0,eff^ = k^0^.

For a quasi-reversible electrochemical reaction, the unit-less kinetic parameter $\Psi$ is a function of the heterogeneous standard rate constant k^0^. $\Psi$ is also expressed as an empirical function of the peak-to-peak separation ($\Delta E_{p}$). Both relationships are expressed in equation (2):

$$\Psi=k^{0}\left[ \frac{\pi DnF}{RT} \right]^{-1/2}v^{-\frac{1}{2}}=\frac{-0.6288+0.0021 (\Delta E_{p}\cdot n)}{1-0.017 (\Delta E_{p}\cdot n)} (2)$$

where the following terms and units apply:

$k^{0}$= heterogeneous standard electron transfer rate constant in cm s^-1^

$D$ = Diffusion coefficient of the reactant in the electrolyte (here: 7.63 x 10^-6^ cm² s^-1^, “Handbook of Electrochemistry” 1^st^ Ed. 2007, C.G. Zoski)

$n$ = number transferred electrons per reaction (here: 1)

$v$ = the scan rate

$F$ = Faraday’s constant = 96485 A s mol^-1^

$R$ = universal gas constant = 8.314 J mol^-1^ K^-1^

$T$ = absolute temperature (here: 296.15 K)

$\Delta E_{p}$= peak-to-peak separation in a CV scan **in millivolts**

Values of $\Psi$ can therefore be determined from $\Delta E_{p}$ recorded at different scan rates and then plotted against $v^{-\frac{1}{2}}$. The slope of the linear regression is then the term marked red in eq. (2) which contains only k^0^ and known constants. Division of the slope by the constants then delivers k^0^.


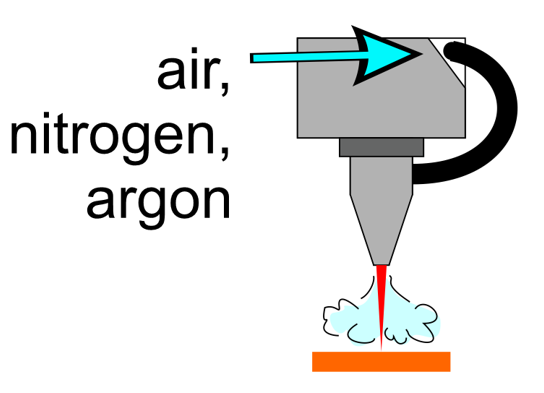


Fig. S 1: Sketch of gas flow into chamber through air-assist


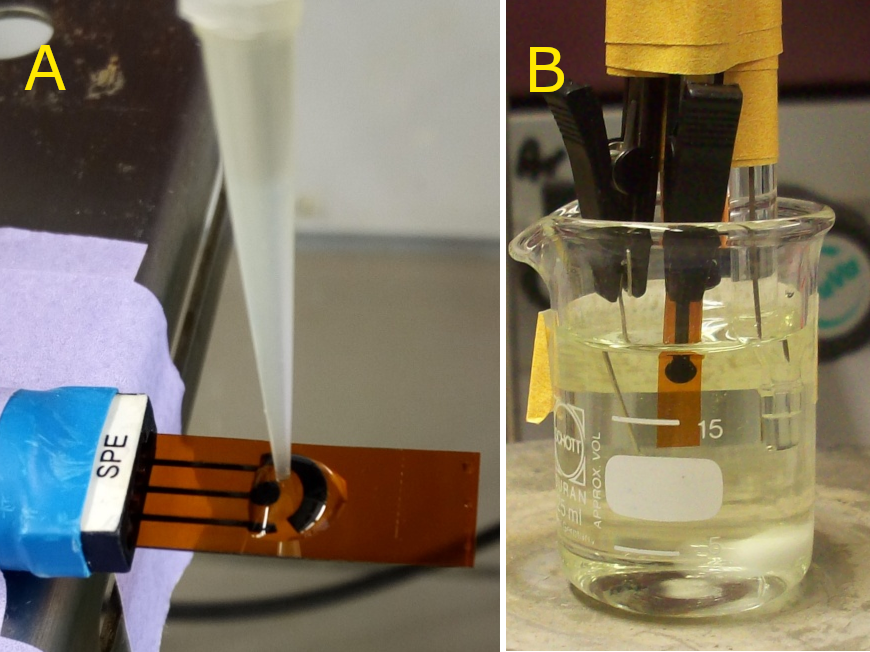


Fig. S 2: Photos of electrochemical cell setup using either a sitting droplet in combination with the 3-electrode design (A) or in a beaker (B). The pipette tip contacting the droplet in (A) is not dispensing solution but is instead the agarose-gel filled second junction of a Ag/AgCl reference electrode (not seen in the foto).


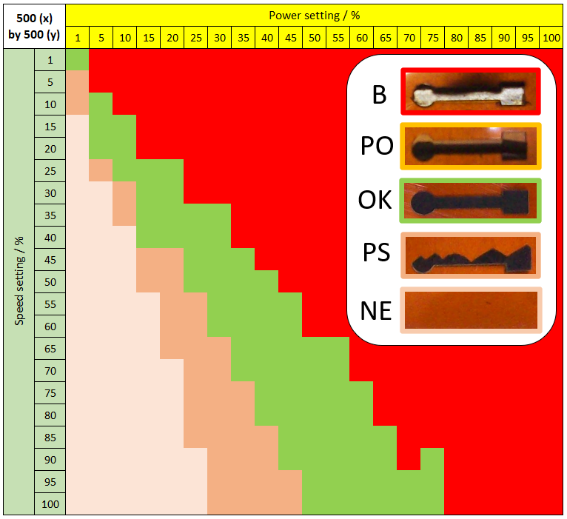

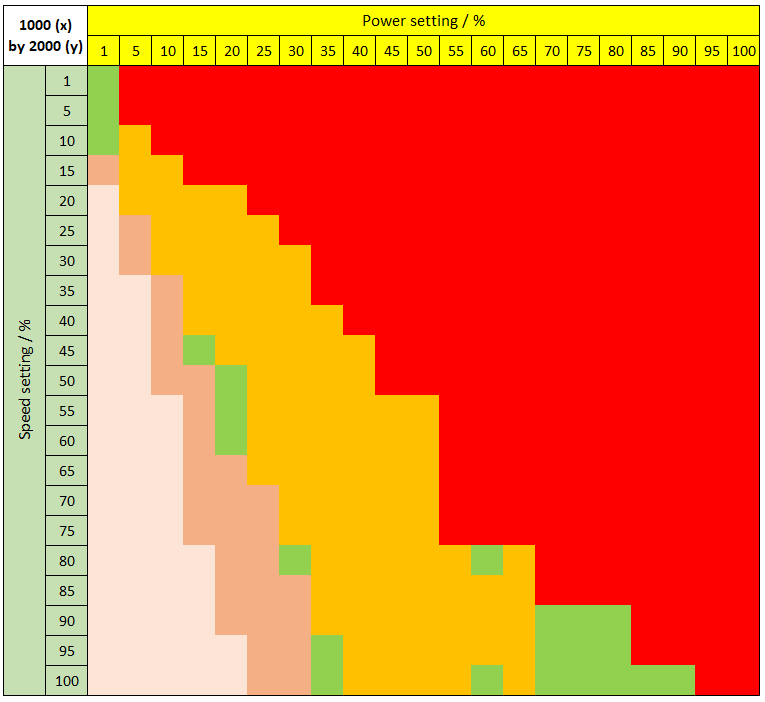


Fig. S 3: Heatmaps of electrode outcome vs. power- and speed settings at pulse densities of 500x500 and 1000x2000 (color code as indicated in inset: green = ok, darker brown = partial scribing (PS), lighter brown = no effect (NE), orange = LIG peeled off from substrate (PO), red = laser burned through substrate (B))


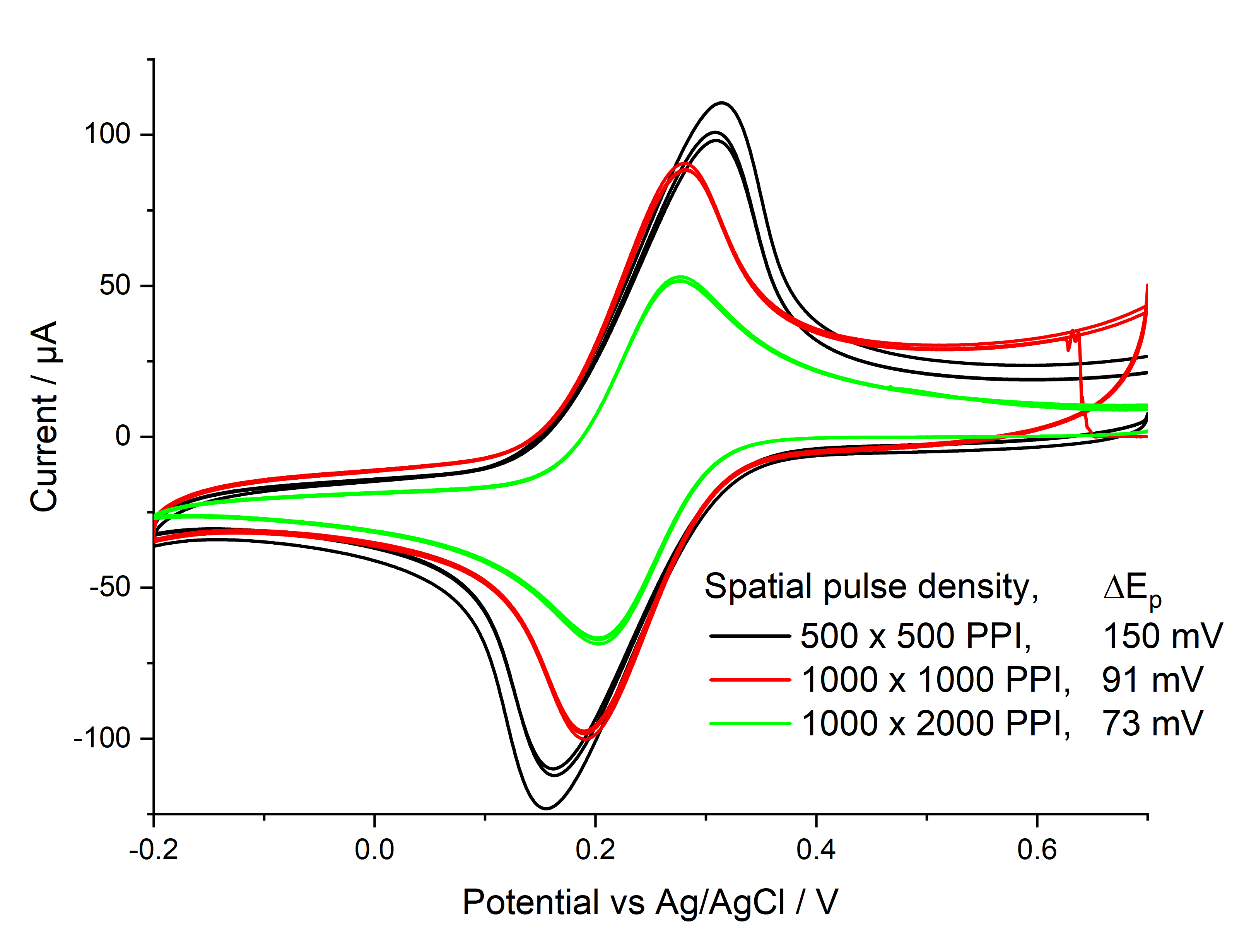


Fig. S 4: CV of 5 mM K_3_[Fe(CN)_6_] on LIG electrodes made at different spatial pulse densities. The best power/speed combinations from each survey were chosen for this comparison. They were 15/30, 1/10 and 1/10 (P%/V%) at 500x500, 1000x1000 and 1000x2000 respectively.


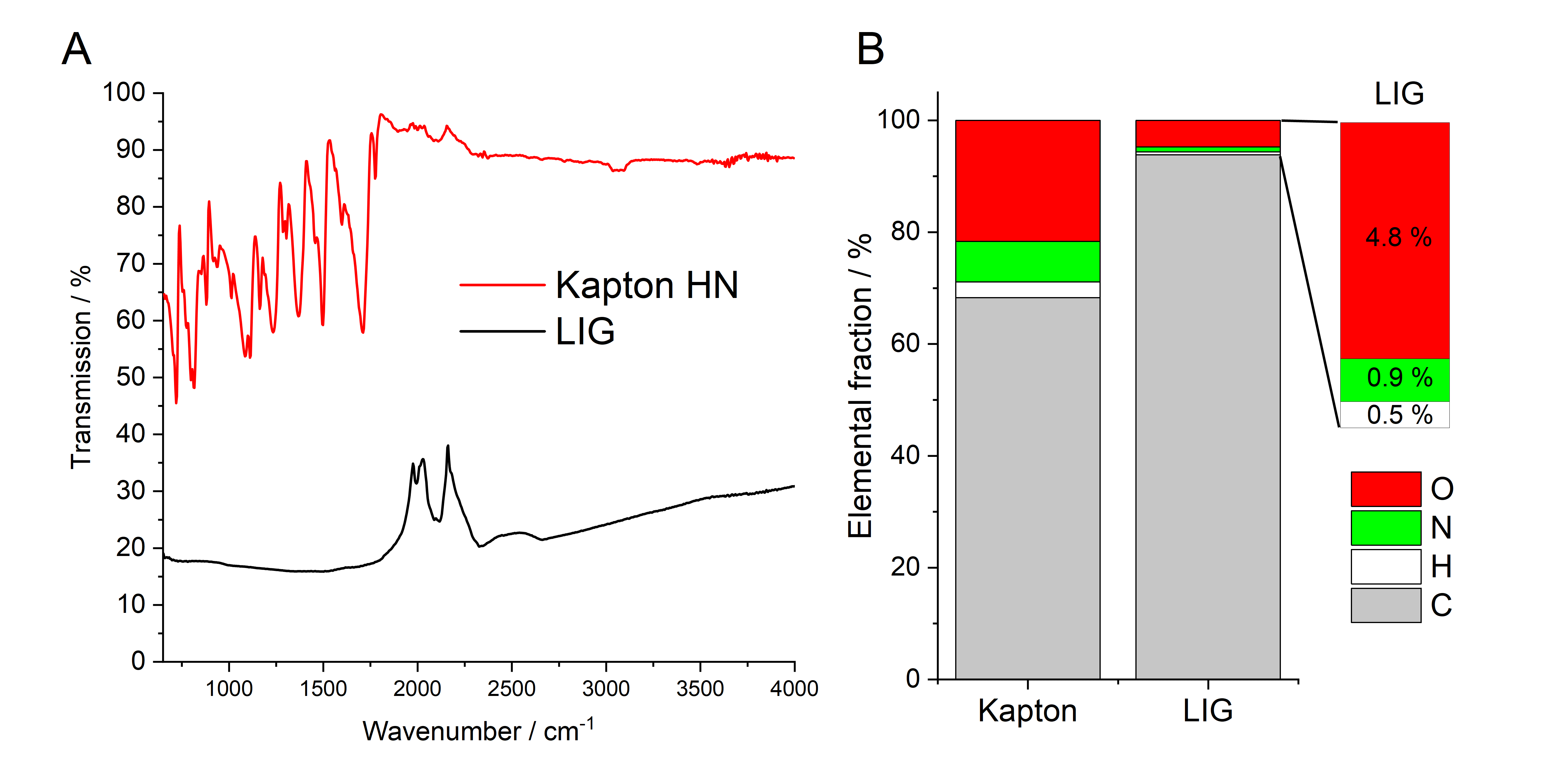


Fig. S 5: a) ATR-FTIR spectra and b) combustion elemental analysis of LIG and Kapton


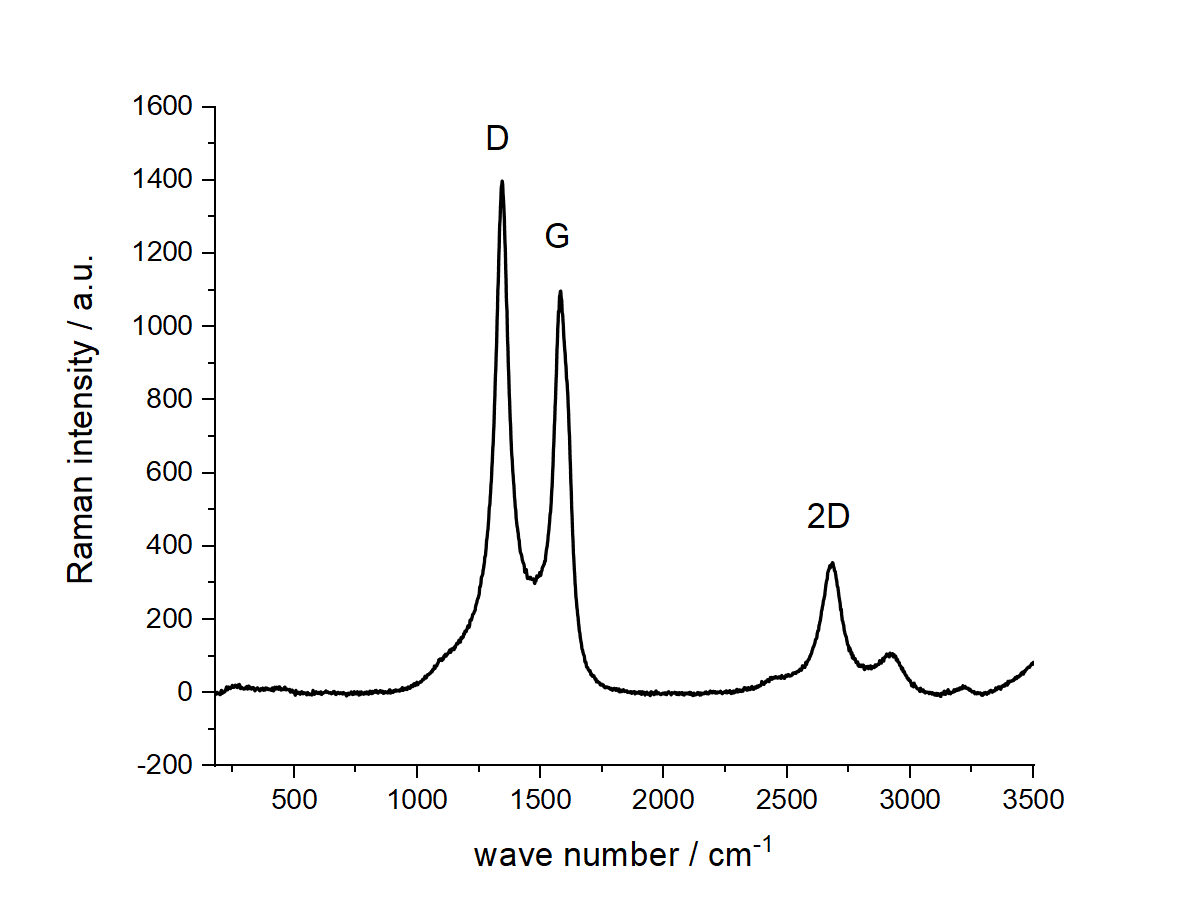


Fig. S 6: Raman spectrum of LIG scribed at 1 % power, 10 % speed and 1000 by 2000 PPI (x by y)

Fig. S 7: XPS spectrum of LIG scribed at 1 % power, 10 % speed and 1000 by 2000 PPI (x by y)


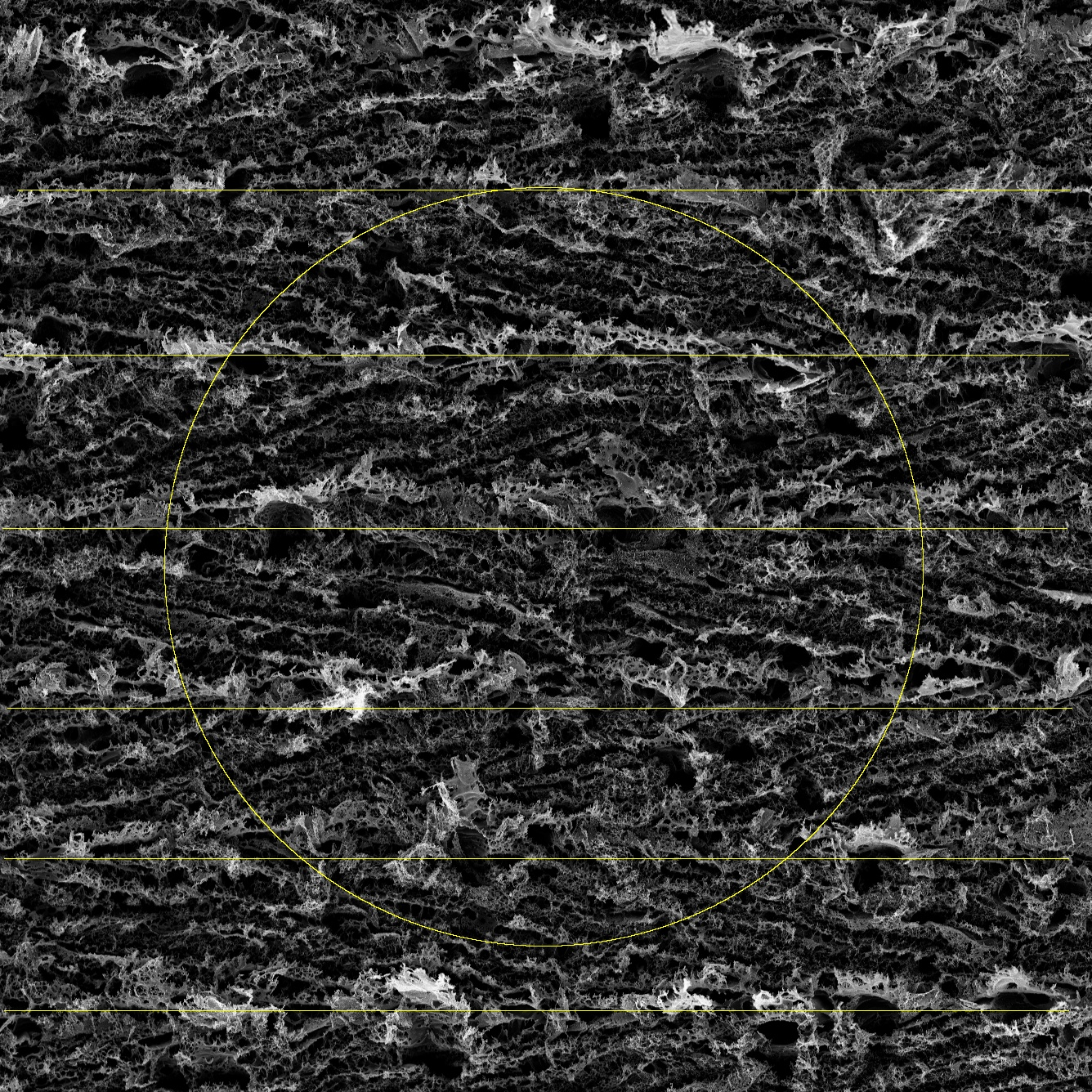


Fig. S 8: SEM image of LIG with emphasized pitch lines (average 25 µm apart) and projected beam diameter (125 µm), settings 1 % power, 10 % speed and 1000 by 1000 PPI (x by y)

Equation for overlap factor:

$$Overlap factor= \frac{\frac{\pi}{4}d^{2}}{pitch_{x}\cdot pitch_{y}}$$

$d$ = beam diameter, e.g. 125 µm

$pitch_{x,y}$ = distance between two pulses in x- or y-direction, e.g. 25.4 µm at 1000 PPI


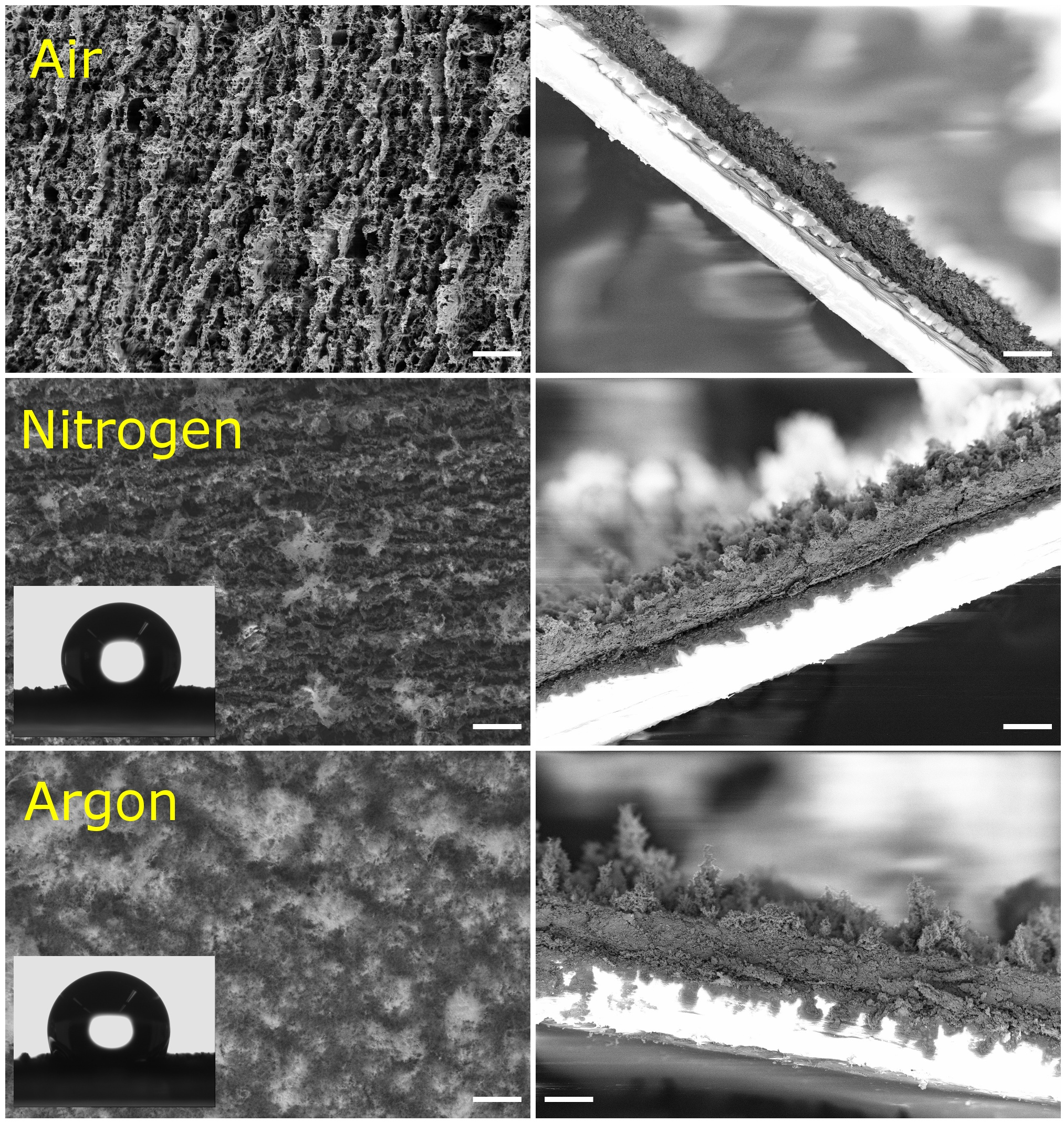


Fig. S 9: SEM images of LIG (1 % power, 10 % speed, 1000 by 2000 PPI (x by y)) scribed while under flow of air, nitrogen or argon; scale bars on left are 10 µm, bars on right are 50 µm

Scribing under argon atmosphere yielded tree-like protruding structures on top of a LIG base. Nitrogen gave a similar less pronounced effect. Surfaces became hydrophobic when scribed under argon and nitrogen (see pictures of water droplets in inset) with approx. contact angles of 150° and 170°, while the surfaces scribed in air remained hydrophilic (perfect spread of water droplet).

The gases were supplied with a flow of approx. 10 L/min through the gas assist system, and thus were directly delivered to the position of the laser beam. Total atmospheric control, however, was not achieved, since the negative pressure generated through the machine’s exhaust system was not fully compensated by the supplied gases and air was sucked from the lab into the non-gas tight chamber.


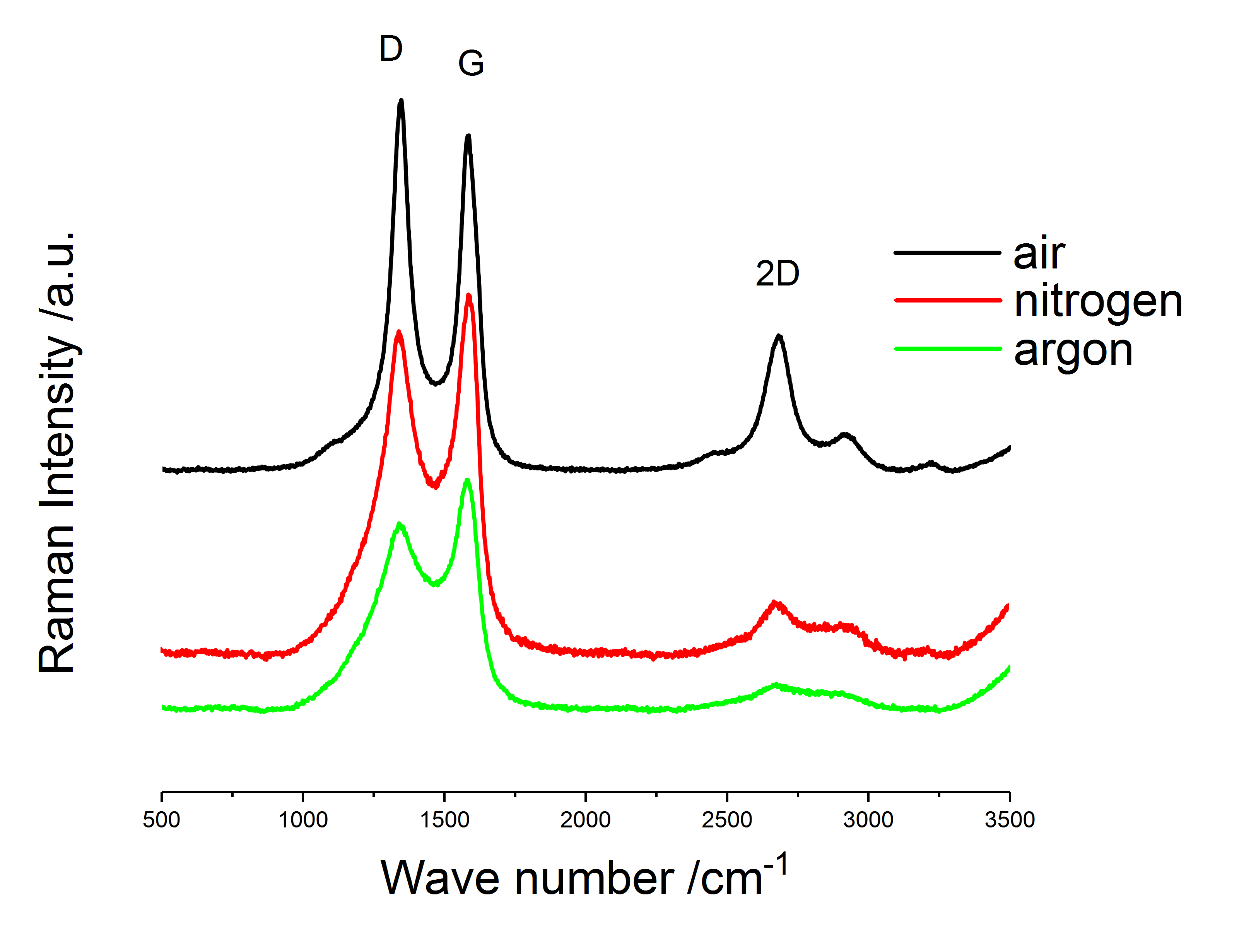


Fig. S 10: Raman spectra of LIG scribed under air, nitrogen and argon atmosphere (1 % power, 10 % speed and 1000 by 2000 PPI (x by y))


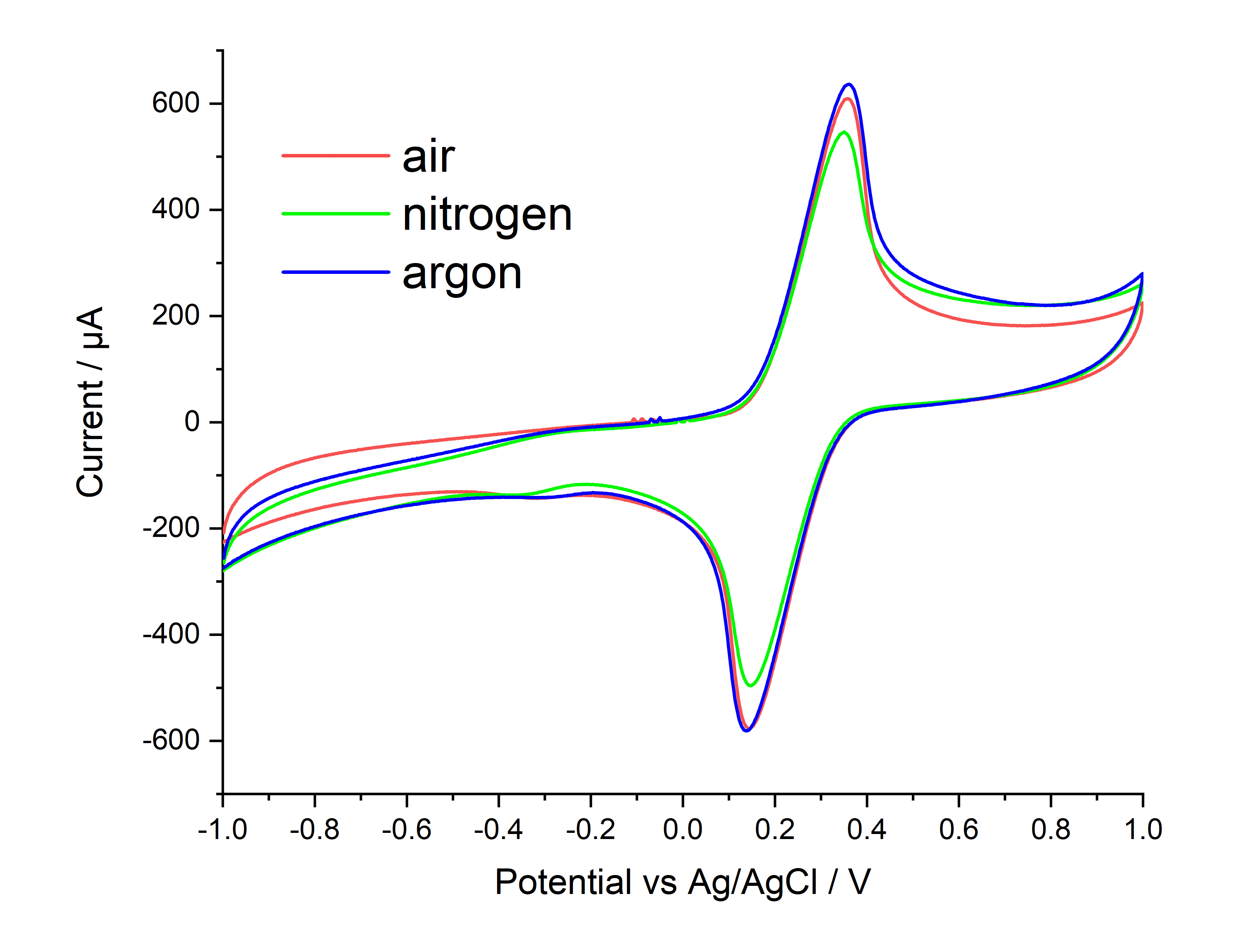


Fig. S 11: CV of ferri-/ferrocyanide (10 mM in 100 mM K2HPO4/KH2PO4, pH 7.4, 100 mM KCl, 100 mV/s) using LIG electrodes scribed under different atmospheres


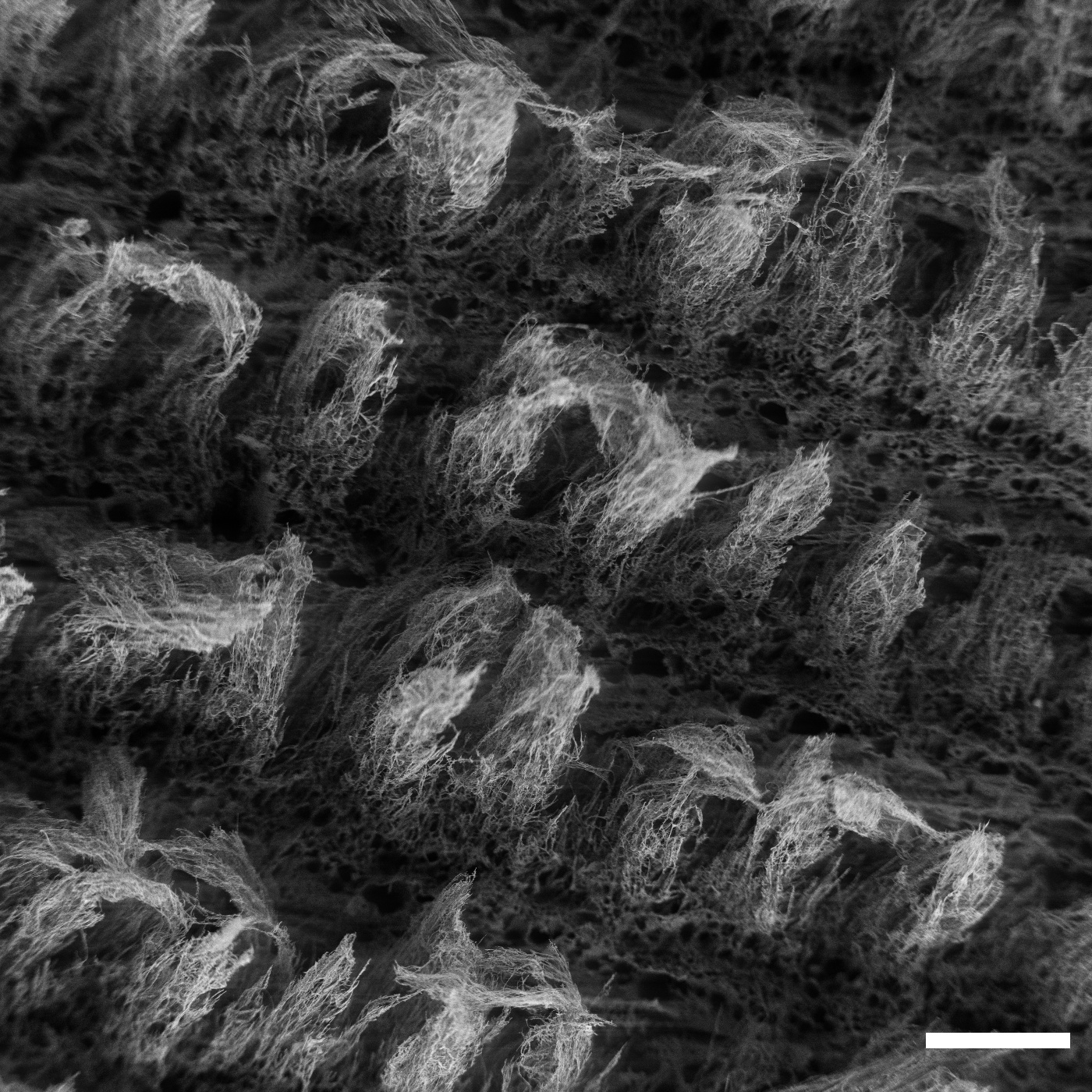


Fig. S 12: SEM image of fibrous LIG created with a pulse density of 500 x 500 PPI (55 % power, 85 % speed), scalebar is 50 µm

Fig. S 13: EIS spectrum of LIG and Dropsens carbon screen printed electrode in ferri-/ferrocyanide


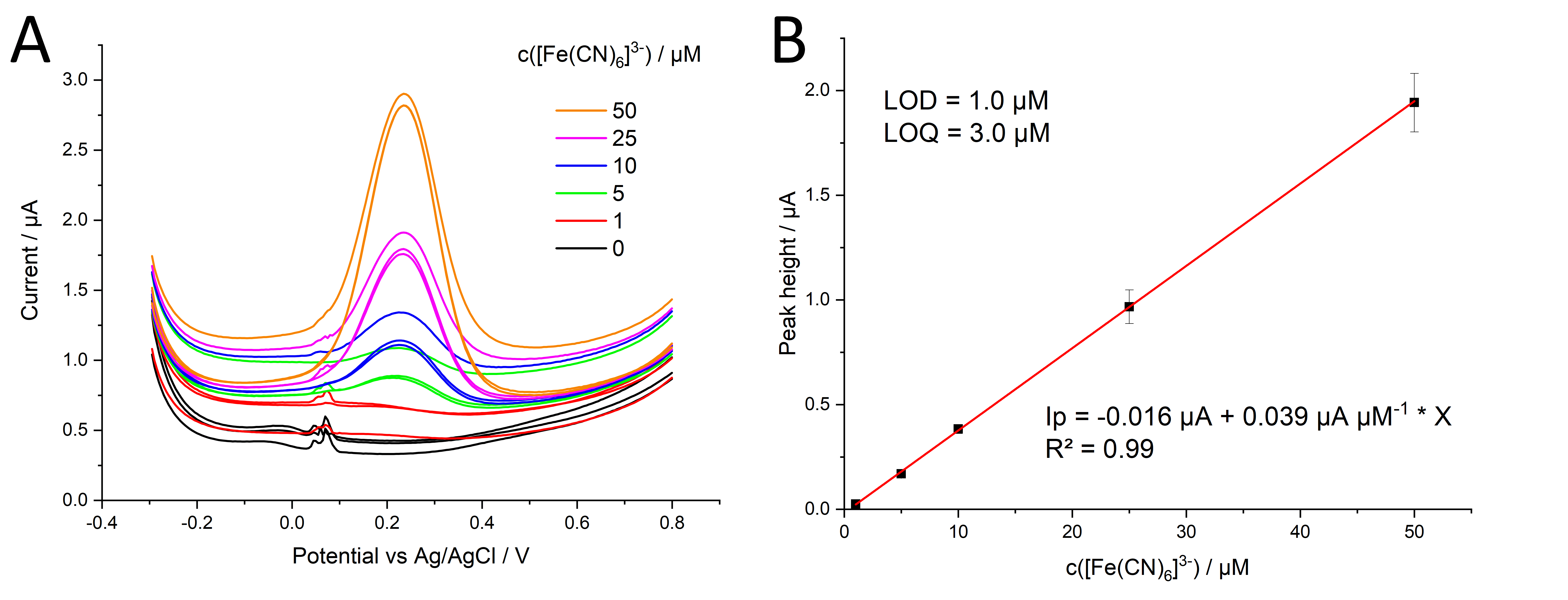


Fig. S 14: Calibration plot of K_3_[Fe(CN)_6_] in PBS on screen-printed electrodes (Dropsens DRP-110, for comparison to LIG); SWV parameters were adjusted to the electrode type: step = 5 mV, amplitude = 80 mV, f = 5 Hz


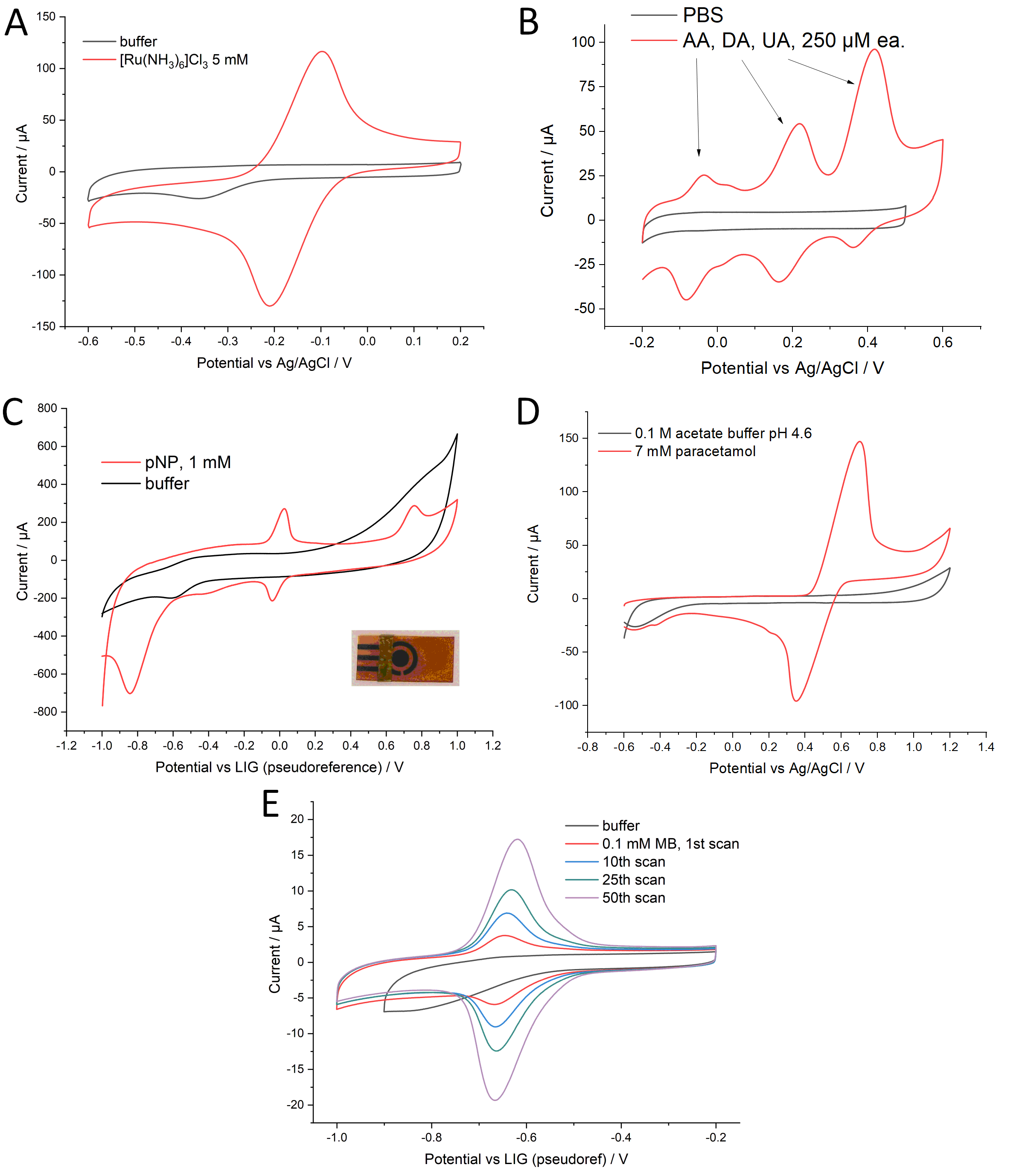


Fig. S 15: Cyclic voltammograms of different chemical species of interest, recorded on LIG electrodes: A) 5 mM [Ru(NH3)6]3+ in PBS; B) mix of each 250 µM ascorbic acid (AA), dopamine (DA) and uric acid (UA) in PBS; C) 1 mM para-nitrophenol in PBS; D) 7 mM paracetamol/tylenol in acetate buffer; E) 0.1 mM methylene blue in PBS, adsorption of MB onto LIG over time indicated by consecutive CV scans. LIG electrodes of the 3-electrode design were used in all measurements.


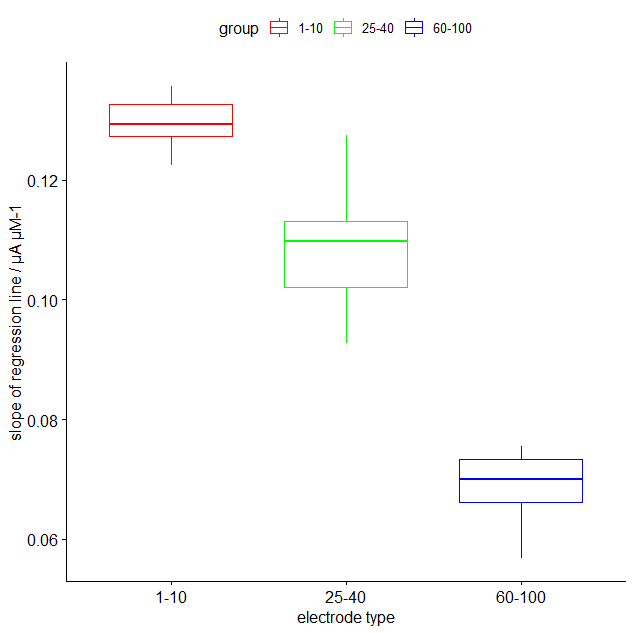


Fig. S 16: Boxplot comparing the sensitivity of K_3_[Fe(CN)_6_] detection (as slopes of the linear regression in the calibration plots in Fig. 4C, F and I). The numbers of electrode replicates were 9, 10 and 5 for the types 1/10, 25/40 and 60/100 respectively.

Tab. S 1: Mean and standard deviation of calibration slopes

|  | slope / µA µM-1 | | |
| --- | --- | --- | --- |
| Type | MEAN | STDEV | n |
| 1/10 | 0.130 | 0.004 | 9 |
| 25/40 | 0.109 | 0.012 | 10 |
| 60/100 | 0.068 | 0.007 | 5 |

Tab. S 2: p-values obtained from one-sided t-tests (H_0_ = difference of the means is zero, H_1_ = difference between means (A minus B) is larger than zero); the null hypothesis was rejected in all cases at p < 0.0001

| A | B | p-value |
| --- | --- | --- |
| 1/10 | 25/40 | 6.10E-05 |
| 1/10 | 60/100 | 8.30E-11 |
| 25/40 | 60/100 | 4.70E-06 |
